# A Comprehensive Review of Floor-Integrated Triboelectric Nanogenerators from Different Perspectives

**DOI:** 10.3390/s26072061

**Published:** 2026-03-25

**Authors:** Sofía Paramio Martínez, Qin Luo, Carolina Hermida-Merino, Jorge Edison Pozo Benavides, José Sánchez del Río, De-Yi Wang

**Affiliations:** 1 Departamento de Ingeniería Eléctrica, Electrónica, Automática y Física Aplicada, ETSIDI, Universidad Politécnica de Madrid, 28012 Madrid, Spain; s.paramio@alumnos.upm.es (S.P.M.); qin.luo@alumnos.upm.es (Q.L.); carolina.hermida.merino@upm.es (C.H.-M.); jorge.pozo.benavides@alumnos.upm.es (J.E.P.B.); 2 IMDEA Materials Institute, C/ Eric Kandel 2, 28906 Madrid, Spain

**Keywords:** triboelectric effect, floors, movement energy, electrical output

## Abstract

**Highlights:**

**What are the main findings?**

- A comprehensive classification of floor-integrated triboelectric nanogenerators is presented, correlating material systems, operating modes, electrical output, and application scenarios.
- A novel FKM–NBR-based triboelectric floor module is experimentally validated, demonstrating stable voltage and current outputs with clear frequency- and position-dependent behavior.
- A maximum power generation of 4 W/m^2^ was obtained.

**What are the implications of the main findings?**

- The comparative analysis provides practical design guidelines for selecting materials and architectures in scalable, durable, and application-oriented triboelectric floors.
- The proposed elastomer-based floor module highlights the feasibility of modular triboelectric flooring for both energy harvesting and self-powered sensing in smart buildings.

**Abstract:**

The harvesting of energy from movements is one of the purposes of triboelectric nanogenerators (TENGs). Among the various devices designed to perform this function, floors are one of the primary ones, as they do not need to be individually fitted to each subject and can be manufactured and installed on a large scale. This work classifies previously published TENG-based floors based on their materials, electrical performance in terms of the voltage, current, and power they produce, and their application in different fields. The materials used have been correlated with other important aspects for floors, such as weather or flame resistance, sustainability, recyclability or biodegradability of materials, and price. The synthesis of the variety of TENG-based floor models, which incorporate novel materials, hybrid technologies, or various functionalities, among other characteristics, can enrich and inspire the reader to enhance the performance of future floor designs based on the triboelectric effect. In addition, a novel triboelectric floor design made of nitrile butadiene rubber (NBR) and fluorine kautschuk material is presented, along with the electrical power generated when tribolayers are in contact. For the three floor strips measuring 40 cm long × 4 cm wide and 1 mm thick, electrical current and voltage output was measured, achieving nearly 0.1 W (20 V & 4.5 mA) of electrical power generation.

## 1. Introduction

The increasing demand for energy in modern society, coupled with the urgent need to find clean and sustainable energy sources, has propelled research and development in innovative areas of energy generation. One of these innovative approaches is harnessing the triboelectric effect as a viable source of electricity generation in everyday environments. The triboelectric effect, which generates electrical charge from mechanical energy in the form of friction or contact between materials, offers significant potential for energy harvesting in everyday environments and situations where conventional electrical infrastructure is limited or inaccessible.

The introduction of triboelectric nanogenerators (TENGs) has brought about numerous advancements in harvesting mechanical energy from various tribological processes of daily life in a very short period. They have demonstrated advantages in implementation across various domains, such as the biomedical field [1,2,3], where numerous implantable devices [4,5] monitor patients’ health in real-time [6]. Other examples of its application, categorized according to the following functions, are as follows:

(a) Self-powered sensors

A wide variety of sensors with exceptional sensitivity have been developed for detecting pressures (in some cases down to mPa) [7,8], accelerations [9], velocities [10], magnetic fields [11], gases [12], object motion (such as traffic) [13,14], surface topography [15], and impact energy [16].

(b) Energy harvesting for storage and utilization

Their application in generating electricity from various primary energy sources such as body movements [17,18], wind [19], waves [20,21,22,23], raindrops [24,25], acoustic waves [26], or vibrations [27].

Among the different systems developed to exploit this effect, triboelectric floors have gained particular attention. These surfaces can convert mechanical energy from human motion, such as walking or stepping, into electrical energy, providing a scalable and user-independent solution for powering low-consumption devices or sensors. Unlike portable or wearable harvesters, floor-based systems can operate continuously in high-traffic areas and can be integrated into architectural spaces, pavements, or public infrastructures without altering user behavior.

Due to the flexibility, low cost, simple fabrication, and high efficiency at low frequencies of TENGs, recent advances in devices that collect mechanical energy from movements, whether of the body or objects, have emerged. Apart from those mentioned, other devices of various natures have successfully fulfilled this function, such as wearable TENGs (embedded in clothing [28], shoe soles or insoles [29] and backpacks [17]) or those embedded in pavements like speed bumps [10] or floors. This latter type does not depend on the characteristics of a single individual but can be equally accessible to everyone.

For this reason, this paper compiles, analyzes, and compares harvesting or sensing tile flooring systems capable of generating electricity from mechanical energy, whose operation is based on the use of TENGs. The electrical output of different floor systems or platforms depends on several factors, including the ability to donate or absorb electrons from the materials selected for surfaces subjected to the triboelectric effect. The greater the difference in electro-affinity between the two [30,31], the more electricity is collected. Beyond electrical aspects, other factors such as environmental resistance, recyclability or biodegradability of materials, and cost have been considered. Subsequently, these triboelectric floors are classified according to their intended applications, distinguishing their functionalities and operating modes as either energy harvesters or sensors. Also, the mode of operation governing the tiles or floor systems, whether by contact–separation, lateral sliding, single electrode, or independent triboelectric layer, is considered. In addition, a novel triboelectric floor design based on FKM and NBR tribomaterials is presented, and an increase in the electrical power with the floor surface is demonstrated, reaching a new maximum power efficiency in triboelectric floors.

## 2. Different Materials Used in Triboelectric Floors Reported in Literature

The design and performance of triboelectric floors depend strongly on the choice and combination of materials used as triboelectric layers, electrodes, substrates, and structural components. These materials determine not only the electrical output but also properties such as flexibility, durability, sustainability, and cost. Based on the literature, triboelectric floors can be broadly classified according to the main material family employed: metallic/porous structures, cellulose- or wood-based materials, polymer-based composites, and hybrid or cementitious composites.

### 2.1. Metallic and Porous-Based Triboelectric Floors

**Figure 1:**
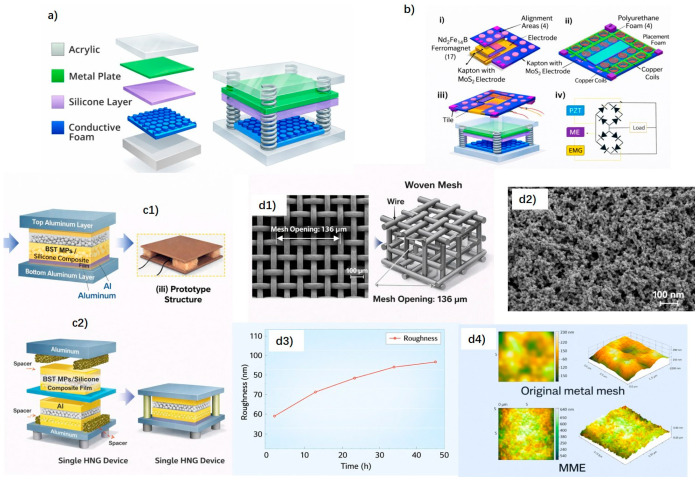
(**a**). Multifunctional triboelectric nanogenerator based on porous micro-nickel foam to harvest mechanical energy [32]. (**b**) Electromagnetic–triboelectric–hybrid energy tile for biomechanical green energy harvesting [33]. (**c1**) Metal–polymer composite-based triboelectric unit using a BST MPs/PDMS film. (**c2**) Enlarged contact–separation unit with spacers [34]. (**d**) SEM images of (**d1**) pristine metal mesh and (**d2**) multi-scale metal mesh electrode (MME) after dealloying, (**d3**) surface roughness evolution with dealloying time, and (**d4**) AFM topography comparison between the original mesh and the MME [35]. AI was used to modify the original figures.

Zhang et al. (2015) [32] reported one of the earliest triboelectric nanogenerators based on porous metallic materials for walking-energy harvesting. A micro-nickel foam (PMNF), serving simultaneously as a tribopositive layer and electrode, was integrated with a flexible PDMS layer to form a TENG operating in combined sliding and contact–separation modes (Figure 1a). Owing to its porous structure, mechanical robustness, and high surface area, the PMNF-based TENG effectively harvested energy from human footsteps and ambient vibrations, demonstrating the feasibility of metallic porous materials in triboelectric floor applications.

Building on metal-based designs, Islam et al. (2020) [33] developed a hybrid floor-tile energy harvester combining a TENG and an electromagnetic generator (EMG) to improve overall output performance. In the triboelectric unit, aluminum electrodes paired with a Kapton-based tribonegative layer enhanced by MoS_2_ coating increased the surface charge density (Figure 1b). The integration of TENG with EMG leveraged the high voltage output of the triboelectric component and the higher current generated by electromagnetic induction, enabling more efficient biomechanical energy harvesting from walking. This work highlights the potential of metal-based triboelectric systems in large-area floor-mounted energy harvesters.

More recently, Paranjape et al. (2023) [34] proposed a metallic-based triboelectric floor concept by integrating multiple consecutively connected hybrid nanogenerators into a floor system (MCHCFS). The device employed an aluminum electrode in contact–separation mode against a BST microparticle-filled PDMS composite layer as the triboelectric counterpart (Figure 1c). The composite layer exhibited enhanced electrical output, reaching approximately 280 V and 8.5 µA, with a surface charge density of 90 µC m^−2^. This multistage floor design demonstrated how aluminum electrodes combined with functional composite layers can simultaneously enable energy harvesting and motion sensing in floor-integrated systems.

From the perspective of electrode architecture, Li et al. (2021) [35] reported a multi-scale metal mesh electrode (MME) designed to enhance triboelectric performance by increasing the effective contact area. The MME was fabricated via an alloying–dealloying process, producing metal wires with microscale mesh structures and nanoscale surface roughness (Figure 1d). When paired with PDMS and operated in contact–separation mode, the MME-based TENG achieved an output voltage of 175.77 V at 4 Hz under a force of 15 N, with a power density of approximately 0.85 W m^−2^. Although demonstrated primarily for respiratory monitoring, this electrode design strategy is directly relevant to floor-integrated TENGs, where mechanically robust metallic electrodes and stable electrical connectivity are essential.

Collectively, these studies indicate that introducing porosity, hierarchical structures, or hybrid configurations into metallic components is an effective strategy for improving both electrical performance and mechanical durability in triboelectric floor systems.

### 2.2. Cellulose- and Wood-Based Triboelectric Floors

Sustainability concerns have driven growing interest in using natural, biodegradable, and renewable materials such as cellulose and wood.

**Figure 2:**
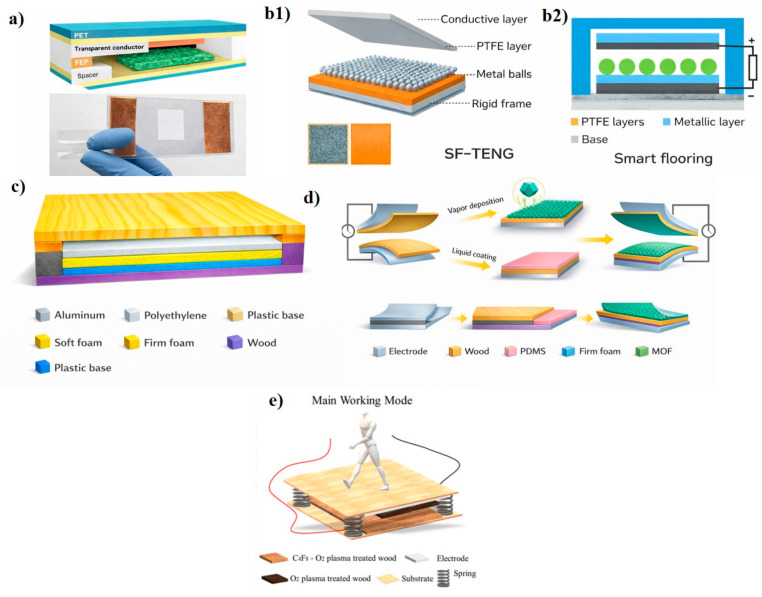
(**a**) Triboelectric nanogenerators and power boards made from cellulose nanofibrils and recycled materials [36]; (**b**) Smart floor with integrated triboelectric nanogenerator as energy harvester and motion sensor. (**b1**) 3D structural point of view; (**b2**) Sectional view. [37]; (**c**) Natural wood-based triboelectric nanogenerator for self-powered sensing in smart homes and floors [38]; (**d**) Functionalized wood with tunable tribopolarity for efficient triboelectric nanogenerators [39]; (**e**) Scalable and sustainable wood for efficient mechanical energy conversion in buildings via triboelectric effects [40]. AI was used to modify the original figures.

In 2016, Yao et al. proposed [36] a TENG based on a cellulose nanofibril (CNFs) film paired with fluorinated ethylene propylene (FEP) as the tribonegative material, as schematized in Figure 2a. By employing a wood pulp oxidation method, the natural cellulose incorporates numerous oxygen atoms, transforming it into a material with an affinity for easily releasing electrons (positively charged) [30]. Both layers are separated by spacers and bonded on their non-contacting faces to two layers of ITO-PET substrate, which also function as electrodes. The transparent TENG is integrated within a fiberboard made from recycled cardboard to test its electrical output and the possibility of using it as sustainable triboelectric large-scale boards or floorings based on wood/fiber.

Other studies have explored the use of wood as the structural and triboelectric material. In 2017 [37], Z.L. Wang and coauthors presented an intriguing floor model based on TENGs. It consists of shallow wooden boxes serving as floor slabs. The floor collects energy in two different ways. In one mode of operation, the wood of the floor itself and the objects moving over it, such as shoe soles, balls, or adhesive tape, act as electronegative and electropositive triboelectric pairs. To induce the triboelectric effect, two layers of materials have been introduced: one of PTFE and another of PTFE upon which aluminum balls, shown in Figure 2b, are at rest. This setup allows energy harvesting when vibrations cause the balls to bounce. Surrounding these PTFE layers there are two aluminum electrodes, each connected to the negative and positive probes for the experiments. The upper electrode, in contact with the upper layer of wood, operates in both modes.

In 2020, Hao, S. et al. developed a W-TENG (wood-based TENG) [38] based on New Zealand pine wood (after testing six different types of wood) as the tribopositive material. This type of wood had a high number of stomata, indicating its ability to generate and absorb electrons. In this case (Figure 2c), the nanogenerator functions by introducing sponges, creating a gap between the wood and a PTFE triboelectric layer (chosen for its high electron-absorbing capacity) as the tribopositive material, which in turn is bonded to a copper electrode, another sponge layer, and a PMMA support.

Further advances were achieved through surface modification of wood. Sun et al. (2021) [39] enhanced the triboelectric performance of wood by growing a zeolitic imidazolate framework (ZIF-8) on its surface, significantly increasing its tribopositive behavior, while PDMS-modified wood acted as the tribonegative layer (Figure 2d). In a complementary study, Sun et al. [40] employed plasma treatments to selectively tune wood surfaces toward tribopositive or tribonegative characteristics without affecting bulk properties (Figure 2e). These strategies demonstrate that surface engineering is an effective route to transform wood into high-performance, scalable triboelectric floor materials.

Mappoli, S. et al. [41] fabricated a fully functional TENG device with 3D multimaterial printing techniques, consisting of positive and negative triboelectric layers, current collectors and a supporting substrate. Nylon 6 and carbon/polyvinylidene fluoride (C/PVDF) filaments are used for positive and negative triboelectric layers, respectively, and conductive carbon/polylactic acid (C/PLA) filament was selected for both current collectors. Wood/PLA was selected for both the top and bottom supporting layers. To demonstrate the high applicability of this triboelectric floor, the MMP-TENG was integrated with electronics, and a smart footstep monitoring system was developed.

Overall, cellulose- and wood-based triboelectric floors combine sustainability with structural versatility, while surface modification strategies are key to further improving their electrical performance and scalability.

### 2.3. Polymer-Based Composites Triboelectric Floors

**Figure 3:**
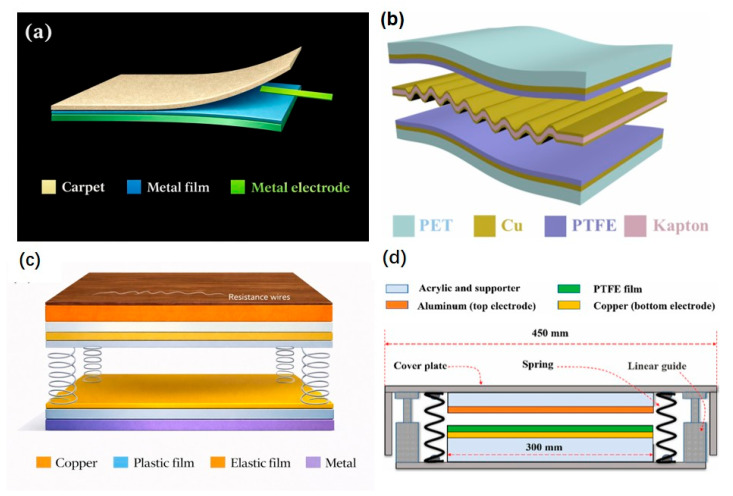
(**a**) From triboelectric nanogenerator to self-powered smart floor: A minimalist design [42]. (**b**) Walking energy harvesting and self-powered tracking system based on triboelectric nanogenerators [43]. (**c**) Triboelectric nanogenerator self-heating floor—possibility of achieving intelligence in architecture [44]. (**d**) Triboelectric energy-harvesting floor tile [45]. AI was used to modify the original figures.

Flexible polymeric layers remain a common choice in triboelectric flooring because of their adaptability, cost-effectiveness, and high charge affinity. In 2017, Ma. J. et al. presented [42] another floor model based on an FS-TENG (flexible single-electrode TENG), which uses a commercial PVC pavement layer bonded to a copper film. A copper electrode is situated between both triboelectric layers. All parts are indicated in Figure 3a. This electronegative PVC layer, when rubbed with rubber (the main material of soles), acts as the electropositive triboelectric layer in single-electrode mode. When the triboelectric layers are separated, electrons flow from the copper electrode to the ground because the copper film is positively charged, and when they approach each other, the electrons flow back to equilibrate.

In 2020, Yao et al. [43] presented a μ-TENG (undulated TENG, see Figure 3b) to collect energy from walking steps in public areas. The TENG consists of a sandwich structure in which two layers of PTFE, with outer faces coated with copper electrodes and a PET substrate, envelop a wavy elastic structure of Kapton, also coated with copper on both sides. The back Cu electrode was deposited on the unmodified surface of a PTFE film via magnetron sputtering. The wavy configuration of the Kapton film was achieved by fixing it with steel rods and heating it in an oven for 4 h at 100 °C. Later, copper foils were applied to both sides using electron beam evaporation. This wave-shaped central part acts as a tribopositive layer, electricity collector electrode, and separator with the tribonegative layers of PTFE. To increase the electron-absorbing capacity of the PTFE, nanowires were constructed on the PTFE surface through deep reactive ion etching (introducing a gas mixture containing O_2_, CF_4_, and Ar into the plasma chamber for 15 s).

In 2022, D. Jiang et al. [44] introduced a vertical contact-separation TENG wood floor capable of self-heating and energy storage functions. The materials selected for the contact-separation operating mode were dielectric and commercially available. In this case, as can be seen in Figure 3c, Kapton (polyimide) was used for the electron-donating layer, and FEP (fluorinated ethylene propylene) with the formula (C_3_F_6_·C_2_F_4_)_x_ was used for the electron-accepting layer, an electronegative material with a high fluorine content to increase charge transfer. The reverse side of both triboelectric layers has been coated with double-sided copper tape electrodes. These electrodes are further coated with layers of ABS (acrylonitrile butadiene styrene), which act as a substrate for both triboelectric layers, separated by springs. It is presented as a TENG that can be implemented in self-heating wooden furniture or floors, using oak wood as packaging material. Beneath the wooden board surface, an electric heating wire made from nickel-chromium alloy was coiled to generate heat through the Joule effect.

P. Thainiramit and others [45] also published a triboelectric energy-harvesting floor tile in 2022. Its easy-to-fabricate structure consists of PTFE film as the electronegative triboelectric material, aluminum foil as the electropositive triboelectric material and the top electrode, and a copper foil under the PTFE as the bottom electrode. A prototype tile is shown in Figure 3d, with two acrylic supports attached to each triboelectric layer, which in turn connect to a base and a cover. It is separated by four springs with a stiffness constant of 29.4 N/mm and vertical linear guides, ensuring total contact between the PTFE film and the Al sheet.

In [46], J. Deng et al. selected four common materials [polytetrafluoroethylene (PTFE), polyethylene terephthalate (PET), ethylene vinyl acetate (EVA), RB] with smart ceramic tiles (SCTs) that integrate electrodes with ceramic tiles using a layer-by-layer temperature gradient sintering method. Square pellet molds with a side length of 5 cm were utilized to prepare the tile samples, yielding a final tile base with a side length of 4.4 cm. To facilitate future commercial production, the electrodes were fabricated using high-temperature silver paste and sintered in air at 850 °C. The results obtained when using an EsP32 IoT chip to send the triboelectrical signals to the cloud suggest that SCTs offer promising applications for home security monitoring within smart homes when individuals entering a house are monitored while stepping on its triboelectric floor.

### 2.4. Cement-Based and Composite Triboelectric Floors

**Figure 4:**
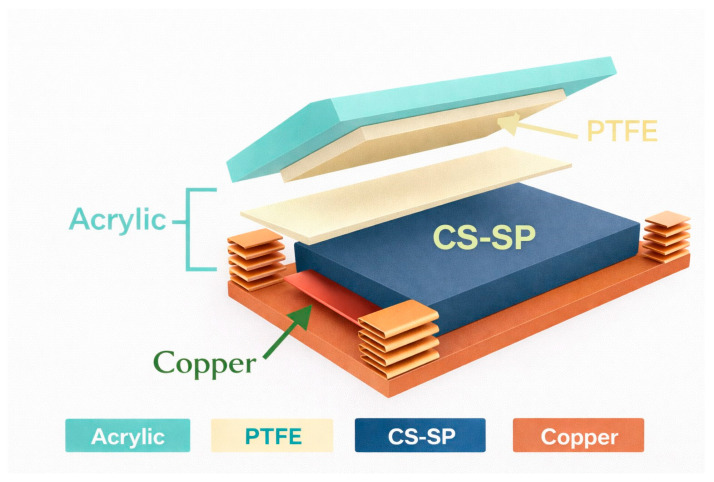
Smart triboelectric floor based on a calcium silicate–carbon composite for energy harvesting and motion sensing applications [47]. AI was used to modify the original figure.

To improve durability and adapt triboelectric systems for architectural or outdoor environments, new composites have emerged that combine traditional building materials with triboelectric functionality. A new floor model proposed by S. Kuntharin et al. [47] was introduced in 2023. It is based on a CS-SP TENG (calcium silicate composites TENG + 0.04 wt% Super P^®^ carbon black). It demonstrates how the addition of conductive Super P^®^ carbon black to a TENG manufactured with calcium silicate composites (one of the main components of cement) increases its dielectric constant and thus contributes to intensifying charge and electrical production. This combination also reduces the appearance of air voids, which changes the surface morphology, improving the compressive strength of the CS composite. A 1.3 times higher compressive strength (46.3 MPa) and a three times greater dielectric constant (82.2) were achieved in the CS-SP @ 0.04% specimen than in the unmodified CS composite. More SP addition caused a reduction of the dielectric constant in CS-SP @ 0.08–0.16%. As represented in Figure 4 the material for the upper triboelectric layer is PTFE, and below the lower triboelectric layer of ‘conductive cement,’ a sheet of grade 304 stainless steel has been used as the current collector electrode. Springs attached to two acrylic molds separate both layers.

In summary, triboelectric floors have evolved from simple metallic and polymer systems to multifunctional and sustainable flooring platforms. Metallic foams and hybrid composites offer high charge density, while natural cellulose and wood ensure renewability and eco-friendliness. Polymer-based designs provide flexibility and ease of manufacturing, and cementitious composites enable robust integration into real architectural structures. This material diversity reflects the adaptability of triboelectric floors to different functional, environmental, and economic requirements.

## 3. Different TENG Floors According to Environmental, Recyclability and Cost Factors

### 3.1. Humidity Resistance or Flammability

The flexible and durable walking energy harvesting device presented in [43] is described as viable and effective regardless of the time of day, season, climate, or weather conditions. Its adaptability makes it an optimal choice for harvesting energy from bustling pedestrian zones.

The electrical output of the wood-TENG proposed in [40] decreases over time due to the deterioration of the plasma treatment with air. Furthermore, its electrical performance under varying relative humidity (RH) levels was examined. The results showed that RH levels between 30% and 50% had minimal effects, whereas humidity exceeding 60% or even 70% resulted in noticeable decreases in output values. For practical applications, the TENG integrated into a self-powered wooden flooring system would be enclosed and shielded from environmental factors, ensuring consistent and long-lasting electrical output.

The self-heating floor [44] system is presented as safe because the value of the current produced is very small (the lowest among all the TENGs discussed in the present review; see Table 1). To increase its firmness and insulation, it is also covered with insulating tape.

In [46], the relative humidity considered appropriate for domestic living usually ranges between 40% and 70%. Within this range, the open-circuit voltage of the SCTs declined from 18.9 V (40% RH) to 1.8 V (70% RH), demonstrating considerable sensitivity to humidity. Although there was a reduction in open-circuit voltage, output power generated could be high enough for sensing applications, as signals in mV are easily detected by any data acquisition system (DAQ).

The electrical output generated by applying impact force on the triboelectric floor tile [45] is tested in Thailand with an oscilloscope at a room temperature of 25 °C and a relative humidity of about 70% in the laboratory. From this, it is deduced that the TENG output may not be affected by humid environments. Recent comprehensive reviews on humidity-resistant TENGs have highlighted various strategies, including encapsulation techniques, hydrophobic surface modifications, and material selection, to maintain stable electrical output under varying humidity conditions [48], providing valuable frameworks for evaluating environmental durability in floor-integrated systems.

### 3.2. Renewability, Biodegradability or Recyclability

TENGs are paving the way as useful technologies toward a ‘net-zero emissions’ future [49]. This fact is not an excuse to overlook their environmental impacts. In most cases, non-biodegradable materials are used as the triboelectric layer to achieve higher electrical output. However, not only synthetic materials can form suitable TENGs for application in floors. Below are some examples.

**Figure 5:**
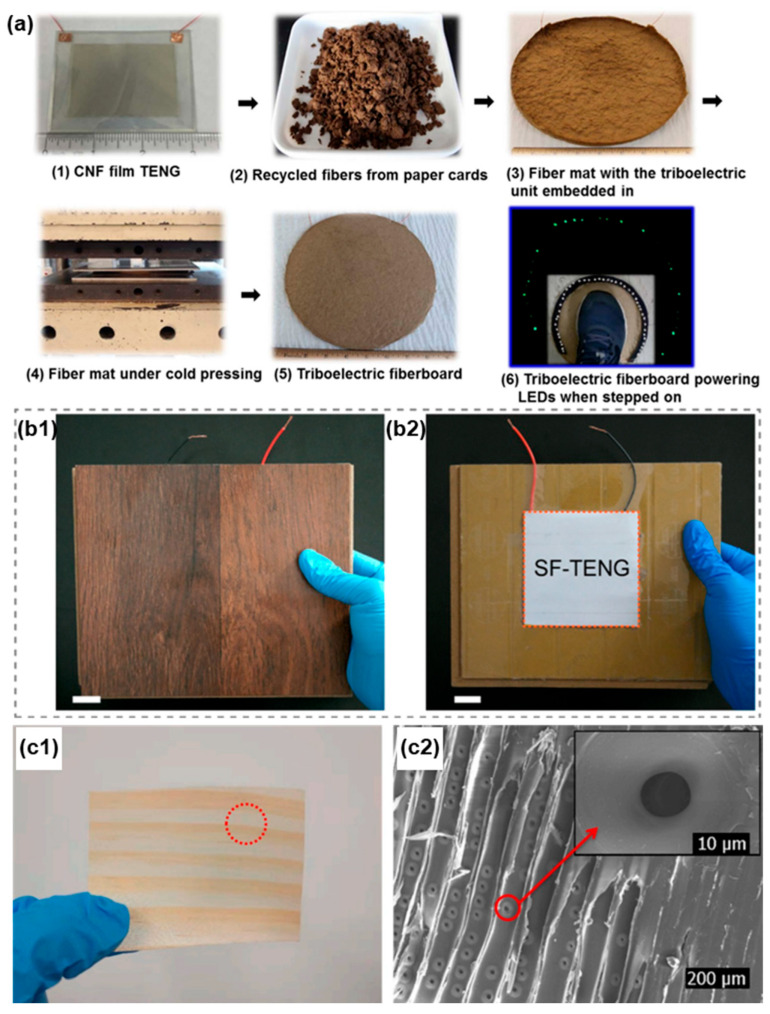
(**a**) Manufacturing process of CNF-based TENG fiberboard [36]. (**b**) Photograph of the (**b1**) front side and (**b2**) back side of the smart floor [37]. (**c**) The structure of natural wood-assembled TENG. (**c1**) The surface topography of New Zealand pine. (**c2**) SEM images of the morphology of New Zealand pine [38]. AI was used to modify the original figures.

The TENG manufactured with CNF [36] as the electropositive triboelectric layer is made from the most abundant natural polymer on Earth: cellulose. In addition to being a biodegradable material, the resulting highly transparent and flexible film, in combination with FEP, achieves performance values (details in the Energy Harvesting section) comparable to those of other pairs of synthetic polymers such as Kapton-PET [50]. The CNF-based TENG was integrated with a fiberboard composed of recycled cardboard fibers through a chemical-free cold pressing technique. The resulting power board showed great recyclability and excellent integrity against mechanical impacts, making it suitable for use as flooring. This process is depicted in Figure 5a.

Other wood-based TENG floors [37,38,39,44] can function as both smart and energy-harvesting floors. Wood is an appealing material due to its sustainability while maintaining important characteristics such as strength and low weight. The SF-TENG [37] utilizes everyday triboelectrically positive chargeable materials, such as balls, shoe soles, clothes, etc., rubbed against the floor surface shown in Figure 5b. This friction between the floor and everyday objects takes place without compromising the stability and flexibility inherent in wood floors. The W-TENG [38] is an environmentally friendly, renewable, sustainable, and easily fabricated wood-based triboelectric nanogenerator because pure, natural, biodegradable and pollution-free wood is used as the triboelectric material. It includes New Zealand pine wood as the tribopositive layer because of the large number of stomata, indicated in Figure 5c, in its morphology. In the FW-TENG [39] and another wood-TENG [40], both layers are made from wood, but they are respectively modified to become electron-donating and electron-attracting (see Materials), achieving a higher electrical output. This method is noted to reduce environmental impact compared to TENGs that simply combine wood with another triboelectric material made from non-biodegradable materials (synthetic polymers) [44] to achieve higher electrical production. In the latter case, reference is made to the end of the TENG’s lifespan, during which the copper foil electrode attached to the back of the wooden piece can be easily peeled off to enable recycling.

The fabricated CS-SP TENG [47] aims to differentiate itself from those based on wood by suggesting that while wood is commonly used in interior design and is more durable than many polymers, it requires additional treatment for outdoor use, unlike this TENG. Another constraint mentioned is that modifying its internal structure or intrinsic properties may also prove impractical. The CS-SP TENG is suitable for both outdoor and indoor use, and it is not difficult to modify its microstructure to improve its triboelectric performance. Since it is based on materials that compose cement, its durability and high compressive strength are also highlighted.

### 3.3. Low Cost

The economic viability of triboelectric floors depends critically on material selection, fabrication complexity, and scalability potential [51]. The hybrid EMG-TENG [33] floor tile is considered a cost-effective design that can be easily mass-produced for biomechanical energy harvesting. Given its high energy conversion efficiency and electrical output, it could be profitable when implemented in public spaces where many individuals walk. It is described as a cheaper renewable energy harvester than wind turbines or solar panels.

In the FW-TENG [39], similar electrical values were obtained for 20-ZIF-8@yew (cross-cut), but spruce was chosen to work with because it was more readily available and cheaper in the market. This layer acts as a tribopositive material in contact with another layer of wood coated with PDMS. This method is noted to reduce the manufacturing cost of a floor on a large scale compared to other TENGs that simply combine wood or cellulose with another synthetic polymer to achieve higher electrical production, thus reducing material costs.

The TENG-based floor with plasma-treated wood [40] identified wood as a cheap construction material suitable for large-scale fabrication. Two prototypes were tested with their respective advantages and drawbacks. One advantage of the single-electrode design is that it is cheaper than the contact-separation working mode, as it requires only one electrode and only one triboelectric wood-treated layer.

The costs of the triboelectric energy-harvesting floor tile (TEHFT) based on a PTFE-Al triboelectric nanogenerator [45] and the PMNF-PDMS TENG [32] were estimated to be low.

The CS-SP TENG [47] based on calcium silicate (CS) offers, among other advantages, low cost and feasibility for upscaling and producing composite materials compared to the polymeric materials typically used for TENGs. To enhance its dielectric constant and, therefore, its electrical production, conductive carbon black is used as a filler. It is emphasized that its cost is significantly lower compared to other conductive materials such as gold or silver nanoparticles and carbon nanotubes.

## 4. Applications

The utilization of electricity generated by self-powered floor models disclosed since the creation of TENGs encompasses multiple applications [52]. These applications have been classified into two main groups: those involved in directly powering lights or small electronic devices of various kinds, and those in which TENGs are used as sensors to detect movements occurring on the floor or to perform specific actions in areas such as sports, smart homes, or even healthcare.

### 4.1. Electrical Energy Harvesting

The electric performance of the reasonably designed first TENG presented above [32] was characterized. The open-circuit voltage (V_OC_) and short-circuit current (I_SC_) were measured as functions of input vibration frequencies (1 to 300 Hz) at an invariable amplitude. The maximum values of 187.8 V and 71.9 μA, respectively, were obtained at a vibration frequency of 13.9 Hz, which coincides with the theoretical resonance frequency of the TENG (sensitivity of 0.954 V/Hz and 5.17 μA/Hz). At that frequency, almost 100 LEDs can be lighted up simultaneously. In addition, a working bandwidth from 11.36 to 22.56 Hz was shown. The higher the load resistance, the lower the instantaneous amplitude of current owing to resistive losses, while voltage exhibits an escalating tendency. Consequently, the instantaneous power reaches its peak at a load resistance of approximately 3 MΩ, corresponding to a maximum peak power value of 9.3 mW and a power density of roughly 3.7 W/m^2^. Footfall experiments were conducted to optimize the electrical performance based on the contact–separation behavior of the TENG. At a force close to 500 N, this TENG can produce V_OC_ and I_SC_ values of up to 403 V and up to 0.34 mA, meaning 0.8 V/N and 0.68 μA/N of sensitivity respectively. The greater electrical output observed with footfall in comparison to vibration shaker experiments is primarily attributed to the substantially increased contact surface area resulting from the larger applied force. Some potential applications for this electrical output are proposed as a self-powered floor and as a means to harvest vibration energy from railways, highways, or tunnels in remote areas.

**Figure 6:**
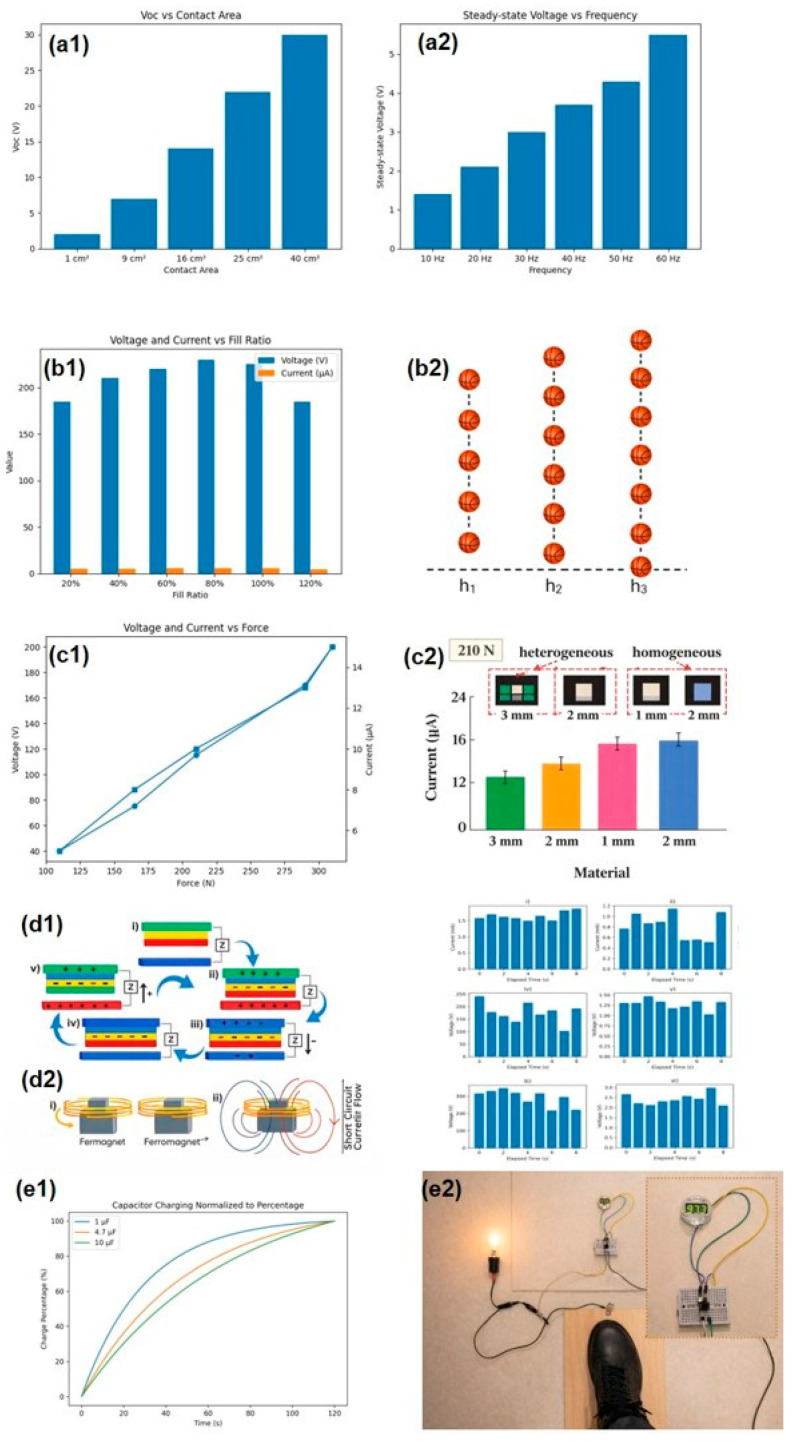
(**a1**) V_OC_ measured from CNF-based TENG with various active surface areas; (**a2**) Voltage measured across a 10 µF capacitor when it was charged by the TENG under different frequencies of mechanical impacts [36]. (**b1**) Open-circuit voltage and short-circuit current of the SF-TENG with different fill ratios (0%, 60%, 80%, 100%, 120%, and 140%). (**b2**) A row of LEDs being lit up by a free-falling basketball at different heights (h_1_ = 40 cm, h_2_ = 80 cm, and h_3_ = 120 cm) [37]. (**c1**) The output current (red) and voltage (black) of the FS-TENG under different impact forces. (**c2**) The current for different test patterns [42]. (**d1**) TENG and EMG individual performance in walking conditions: (i) EMG short-circuit current (244 μA), (ii) TENG short-circuit current, (iii) EMG open-circuit voltage, (iv) TENG open-circuit voltage. (**d2**) Performance of hybrid tile at 60, 90, 120 bpm: (i) short-circuit current, (ii) open-circuit voltage (246 V) [33]. (**e1**) Voltage profile of capacitors (1 μF; 4.7 μF; 10 μF) charged by the W-TENG; the inset shows the equivalent circuit. (**e2**) Electronic watch driven by electricity stored by the WTENG. [38]. AI was used to modify the original figures.

The average peak output values of the CNF-FEP [36] triboelectric nanogenerator, measured with an input impedance of 1 MΩ, indicated a consistent increase in both V_OC_ and I_SC_ with the expansion of the film’s active area (Figure 6(a1)). At a surface area of 40 cm^2^, the highest V_OC_ and I_SC_ peak values reached 32.8 V and 35 µA, respectively, and the maximum power peak value of 0.56 mW was obtained at a load resistance of ~1 MΩ (1.4 mW/m^2^ @ 1 MΩ). Moreover, the TENG’s potential to effectively charge 10 µF, 33 µF, and 68 µF capacitors to supply a DC current was tested by connecting them through a rectification circuit that converted the AC signal. The higher the frequency and the larger the capacitor, the higher the saturation voltage obtained and the longer the time the curves take to reach that voltage value (Figure 6(a2)), respectively. For the 10 µF capacitor, the saturation voltage was reached after approximately 40 s, a typical value for an RC charging. At 10 Hz, the lowest frequency, the TENG successfully illuminated 150 green LEDs in series. Its effectiveness as a viable DC power source was also demonstrated for the TENG-integrated fiberboard, in which one average-weight person’s step lit up 35 green LEDs. With repetitive stepping, peaks of V_OC_ and I_SC_ between 10–30 V and 10–90 µA were produced (this means 1–3 V/Hz and 1–9 μ A/Hz). It was determined that the power board could effectively deliver up to 98% of its triboelectric charge to the external circuit. However, the conversion efficiency from input mechanical energy to output electrical energy was approximately 8.3%. This lower efficiency may be attributed to the predominant storage of input mechanical energy as mechanical deformation, given the inherent rigidity of the fiberboard.

In the SF-TENG [37], energy harvesting is achieved through two modes of operation. Mode I harnesses the energy from the vibration of the balls due to stronger impacts (such as the bouncing of basketballs or the jumping of athletes). Mode II exploits the friction between the wood and objects in contact with the ground (such as shoe soles or a ground ball) during activities like walking, dancing, or playing basketball. Measurements were taken by varying design parameters such as the spacings (2 mm, 4 mm, or 6 mm) between the top and bottom PTFE films that surround the Al balls at different frequencies, and varying the fill rate (0%, 60%, 80%, 100%, and 120%), which is the number of these balls placed into the square frame TENG (Figure 6(b1)). The results were optimal for a spacing of 4 mm at 30 Hz and a 100% fill rate. For example, with the repeated bouncing of a ball, an average production of 364 ± 43 V and 9 ± 1 μA (12.13 V/Hz and 0.08 μA/Hz) was achieved, simultaneously lighting a total of 87 LEDs connected in series (as the bounce height increases, so does the light intensity; Figure 6(b2)). With normal human steps, a voltage of 238 ± 17 V and a current of 2.4 ± 0.3 μA are produced, with the first step around 100 V. This difference is due to the accumulation of charges with each step until reaching the stable saturation voltage.

The floor with the FS-TENG [42] was rubbed with a moving active layer of a latex rubber glove under loading conditions of 210 N and a fixed frequency of 5 Hz. I_SC_ and V_OC_ reached peak maximum values of 7.5 μA and 180 V when the piece of rubber was separated from the floor. This means a sensitivity of 0.85 V/N/Hz and 0.007 μA/N/Hz. The maximum triboelectric charge density was 75.5 μC/m^2^. The maximum instantaneous output power of 0.76 W/m^2^ was achieved with a load resistance of 30 MΩ. Since the PVC flooring is elastic, a higher impact force resulted in a larger contact area (Figure 6(c1)), and vice versa if the force decreases. The respective current and voltage values for 110 N and 310 N were 1.7 μA, 12.2 μA and 85 V, 220 V for current and voltage, respectively, resulting in an almost linear sensitivity of 0.09 μA/N, 0.04 μA/N, 0.77 V/N and 0.07 V/N. The values also increased when the surface area increased. It was observed that the resultant current peak value was slightly higher for a 1 mm PVC layer thickness than for 3 mm, higher with latex rubber as the tribopositive material than with nitrile rubber or wool, and similar for homogeneous and heterogeneous PVC flooring patterns (Figure 6(c2)). Lastly, it can light up at least 250 LEDs by a foot attached to a rubber sheet stomping on the TENG.

The hybrid TENG-EMG nanogenerator [33] was first tested separately; that is, the TENG and the EMG were tested with and without the bridge rectifier. The TENG shows higher open-circuit values than the EMG, while the latter produces a higher short-circuit current (Figure 6(d1)). Under conditions of 2 Hz (120 beats per minute (BPM)) frequency, a load of 63.5 kg, and a foot-tile distance of 0.076 m (3 in), the resultant output values were 4–8 V and 1.25–2 mA for the EMG (0.03–0.06 V/kg/Hz and 0.009 mA/Kg/Hz), and 350–500 V and 20–38 μA for the TENG (2.75–3.92 V/Kg/Hz and 0.157–0.29 μA/Kg/Hz). Testing the device together, to sum the outputs of each one, at different walking frequencies (60, 90, 120 BPM) at a foot-to-tile distance of 0.051 m (2 in), current values between 0.6 and 1.6 mA and voltage values between 200 and 400 V were obtained (Figure 6(d2)). The voltage values are lower than those produced solely by the TENG due to the smaller separation distance. This fact agrees with the output values of the EMG-TENG when jumped on being higher than when simply stepped on, between 0.8–2 mA and 375–875 V compared to 1–1.6 mA and 400–800 V. Despite the tile generating up to 1200 V of direct energy, capacitors lack the capacity to store such high potential. Thus, the device could be more suitable for large-scale deployment, where a battery and generator would be more appropriate. Twenty light-emitting diodes were lit up by tapping with both hands at 4 Hz (240 BPM). The maximum instantaneous power is calculated by multiplying the maximum voltage and current values: 1200 V × 5 mA = 6 W or 1.5 W/Hz. Due to its substantial output, this hybrid tile can be applied as flooring in gyms, footpaths, or public areas, where human energy expenditure is high. Connecting multiple TENG-EMG hybrid tiles in a series circuit offers an ideal means of accumulating power from each tile’s output, enabling large-scale power generation.

The W-TENG model [38] produces a quantity of 220 ± 20 V and 5.8 ± 0.5 μA at a frequency of 2 Hz and a peak velocity of 0.352 m/s, resulting in a sensitivity of 110 V/Hz and 2.9 μA/Hz. An increase in voltage and current output values has been observed with an increase in contact area or frequency. The maximum power density can reach 158.2 mW/m^2^ at a resistance of 50 MΩ. It can light up more than 42 light-emitting diodes by stepping on the floor with a slipper made of EVA (measuring 200 V and 3 μA as peak voltage and current values). Through a rectifier circuit connecting the TENG with a capacitor, the charging curve of capacitors of 1 μF, 4.7 μF, and 10 μF has been tested (Figure 6(e1)). They reached voltage values of 7.4 V, 4.7 V, and 3.2 V in 120 s, as greater storage capacity results in slower capacitor charging. The voltage profile measured experimentally while walking for 90 s with the slipper showed that the 1 μF capacitor reached a charge of 5.8 V. When the energy is stored in a commercial capacitor of 47 μF and reaches 1.5 V, an electronic watch can be powered (Figure 6(e2)).

The u-TENG [43] demonstrated an output voltage of 86 V and an output current of 6.2 μA when subjected to an impact of 500 N at a frequency of 1 Hz (sensitivity of 12.4 nA/N/Hz). When the impact frequency was increased to 5 Hz, the open-circuit voltage remained at 86.0 V, while the short-circuit current rose to 10.8 µA (17.2 V/Hz and 2.16 μA/Hz). After testing the impedance dependence of the fabricated u-TENG, the maximum output power of 0.279 mW was observed at a loading resistance of 300 MΩ. Driven by human walking, the outputs were sufficient to continuously illuminate 110 light-emitting diode (LED) bulbs, with all LEDs activated by a single footstep. Additionally, the real-time dynamic signal profile of the output voltage for an adult man, an adult woman, and a child is presented, showing the increasing tendency of the signal with weight. This study proposed the TENG as a strategy for mechanical energy harvesting in public areas (like subway stations, hospitals, shopping malls, and business streets).

**Figure 7:**
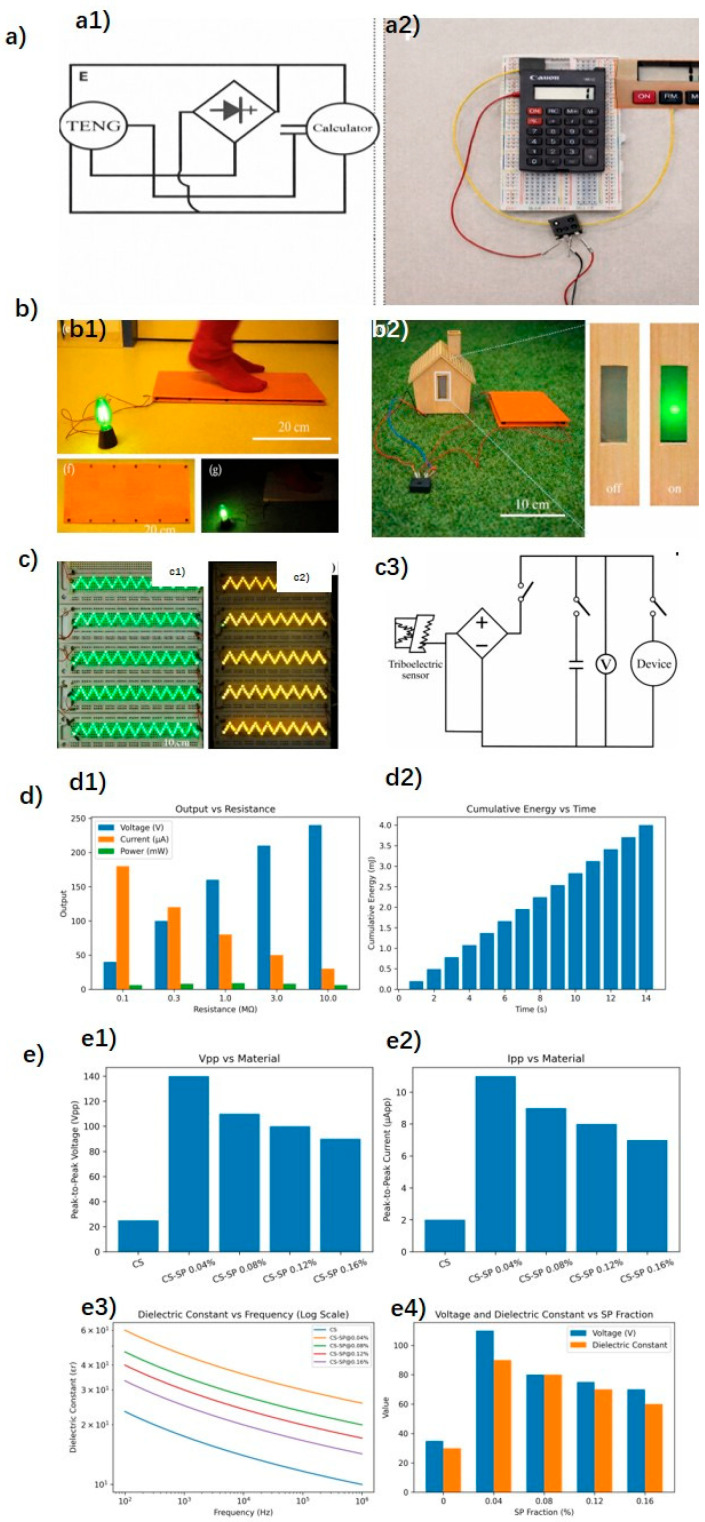
(**a**) Schematic representation of a FW-TENG used to power (**a1**,**a2**) a calculator [39]. (**b**) (**b1**) A W-TENG used to power a smart window of a model wooden house, respectively. (**b2**) A bigger prototype (45 cm × 20 cm) of the W-TENG floor made of eight pairs of plasma-treated wood connected was used to power a household lamp [40]. (**c**) (**c1**) Green and (**c2**) red LEDs powered by the TENG floor; (**c3**) Circuit diagram of the self-powered system for powering electronics or charging energy storage devices [44]. (**d**) Electrical characteristics of TEHFT with a 0.1 mm PTFE film thickness: (**d1**) output voltage, current, and power across resistive loads; and (**d2**) graph of output voltage and accumulated energy across the optimal load [45]. (**e**) (**e1**) Electric output voltage and (**e2**) output current. (**e3**) Dielectric constants of CS and CS-SP@ 0.04–0.16% composites with an inset of dielectric loss measured at frequencies of 102–106 Hz. (**e4**) Plot of TENG output voltage and the dielectric constant of CS-SP composites at various SP concentrations [47]. AI was used to modify the original figures.

The FW-TENG tried multiple material combinations to achieve the highest possible electrical output [39]. The electrical output of spruce wood (cross-coated) modified by ZIF-8 nanocrystals, grown on the wood through a synthesis process explained, was compared with a ZIF-8 modification applied by physical coating. The second method results in less observed nanoscale surface roughness and, consequently, lower electrical output as well. As the size of the ZIF-8 nanocrystals grows in the wood, the electrical output increases with the ratio between the ligand (2-MeIm) and the metal ions (Zn^+2^ cations). Under a force of 50 N, rubbing native radical-cut spruce wood against spruce with different MeIm/Zn^+2^ ratios (1, 5, 10, and 20) and cutting directions (cross-cut, tangential-cut, and radial-cut), the maximum values were 6 V and 0.07 μA for the radial-cut section with a ratio of 20 (sensitivity of 0.0014 μA/N). The increase in output due to the size of ZIF-8 is attributed to the increase in roughness and the tribopositive nature of ZIF-8. Radial cutting leads to higher electrical outputs due to the reduction in pore size and, thus, a larger effective contact surface. Under the same conditions, after trying different tribonegative combinations, spruce radial-cut coated with PDMS also showed the highest electrical output values (18.5 V and 0.25 μA) due to its higher roughness (0.37 V/N and 0.005 μA/N). The final result is a TENG composed of the combination of the most tribopositive and tribonegative parts: radial-cut spruce wood modified by in situ-grown ZIF-8 (2-MeIm/Zn + 2 ratio of 20) and coated with PDMS, respectively. It can generate maximum values of 24.3 V (V_OC_) and 0.32 μA (I_SC_) under 50 N of force (V_OC_ = 0.486 V/N and I_SC_ = 0.0064 μA/N). To maintain the robustness and color characteristics of wood, a thicker layer is used compared to more conventional triboelectric materials. However, it is mentioned that with a thinner thickness, the electrostatic induction effect would be stronger, increasing the electrical output. The electricity generated by FW-TENG can be stored in capacitors for future use. Different commercial capacitors (0.1 μF, 0.47 μF, and 2.2 μF) were charged, reaching voltages of 8.9 V, 2.2 V, and 0.48 V within 30 s. By connecting external resistors (ranging from 0.1 to 120 MΩ), it was found that with an optimized load resistance of 80 MΩ, a maximum instantaneous power of 7.3 mW (or a power density of 10.4 mW/m^2^) was achieved. It is proved that the electricity can also be used to power small electronic devices such as a calculator (Figure 7a) by hand tapping, powered after charging a 0.1 μF capacitor for a few seconds.

As in the previous paper, among different cutting forms (cross-cut, tangential-cut, and radial-cut), the maximum voltage and current values of another wood-TENG [40] also occur for wood cut radially due to its higher surface roughness. For an applied force of 40 N, treatment time and power of 5 min and 60 W, and against radial-cut native wood, the tribopositive layer treated with O_2_ plasma shows much higher maximum values than the tribonegative layer treated with C_4_F_8_ + O_2_ plasma, with voltage values of 1.33 V (0.033 V/N) and a current of 0.024 µA (0.0006 µA/N), compared to 24.7 V for the tribopositive layer treated with O_2_. After testing with various treatment times (from 30 s to 30 min), for the same conditions, the voltages contributed by both layers increased until reaching the maximum at 15 min (2.06 V for the tribopositive and 36.7 V for the tribonegative), after which the voltage decreased because the structure of the first layer was damaged and the surface of the second layer became saturated with atoms. Different treatment powers were also tested (ranging from 20 W to 100 W), with maximums at 40 W for the wood treated with O_2_ plasma (2.67 V and 0.067 V/W) and 0.041 μA (0.001 μA/W) and at 80 W for the one treated with C_4_F_8_ + O_2_ plasma (~39.8 V (0.5 V/W) and 0.46 μA (0.005 μA/W)). A TENG formed by the two optimized wood layers with their corresponding plasma treatment conditions obtains ~2.67 V and 0.041 μA as maximum output values. Commercial capacitors (0.1 μF, 1 μF, and 10 μF) were charged for 118 s (reaching voltage values of 40.6 V, 8.5 V, and 0.7 V, respectively). Varying load resistances between 0.1 and 200 MΩ, the maximum instantaneous power was 18.86 mW/m^2^ for an optimal resistance of 120 MΩ. Increasing the periodic contact force to 180 N and the size of the TENG, maximum values of 227 V and 4.8 µA are produced (sensitivity of 1.26 V/N and 0.026 µA/N). This prototype was tested in the same way as the aforementioned wood TENG, powering household electronics like a household lamp (Figure 7(b1)), a calculator, an electrochromic window (Figure 7(b2–b4)) or liquid crystal displays (LCDs). Another prototype operating in the single-electrode mode was developed, using as flooring only one of the plasma-treated wood layers with a copper electrode attached to one of its faces and objects that friction with the floor surface as the other triboelectric layer. The output electrical values in this mode are not as high (205.9 V under a force of 180 N against a copper foil) and depend on the polarity difference of charge that occurs between the objects on the single triboelectric layer, and with the dirtiness of external objects, it deteriorates earlier. However, it has other advantages such as the absence of deformation of the ground when stepped on.

The self-heating TENG floor [44] (Figure 7(c1))displayed a high electrical output with an open-circuit voltage of up to 240 V and a short-circuit current of 550 nA, which was sufficient to illuminate at least 448 series-connected commercial light-emitting diodes (Figure 7(c2)). Evaluating its storage function, capacitors of 220 nF and 10 µF were charged to 18 V between 2 and 8 s, using a full-bridge circuit with switches as shown in Figure 7(c3). After testing various thicknesses of Kapton and FEP films, namely 25, 50, 75, and 100 µm, the maximum values were as mentioned above for 25 µm and 100 µm, respectively. It is demonstrated how the TENG with these final dimensions can also drive small electronic devices such as a digital clock or an alarm. Other potential applications mentioned include mice, keyboards, mobile phones, or IoT devices.

The influence on electrical performance of parameters such as triboelectric material thickness, gap width, and pressing frequency was tested to characterize the triboelectric energy-harvesting floor tile (TEHFT) [45]. A pneumatic actuator provided the pushing force of 13.04 N and the pulling force of 1.42 N by its return spring. They experimented with 8 thicknesses (0.1, 0.2, 0.3, 0.4, 0.5, 0.8, and 1 mm) of PTFE film (tribonegative material), 5 displacements between the cover plate (2, 4, 6, 8, and 10 mm), and a pressing frequency on the cover plate from 0.5 to 3 Hz. Thinner triboelectric layer thickness resulted in higher output voltage, with the highest peak-to-peak open-circuit voltage being 254 V for a thickness of 0.2 mm and increasing to 333.8 V at a gap width of 10 mm, surpassed by the 0.1 mm thick PTFE at displacements of 0.2 and 0.4 mm. However, the 0.2 mm thick PTFE sheet provided the highest cumulative energy. Moreover, when the TENG was connected to a 1 MΩ load to measure the voltage, the 0.1 mm thick PTFE layer provided the highest voltage output (Figure 7d). To determine which one provided the optimum electrical output, they fabricated a prototype with both thicknesses. Because a square wave was used to simulate the pressing frequency on the test bench, the peak-to-peak voltage values remained stable regardless of frequency. The manufactured tile prototype, with a separation distance of 5 mm between layers (to harvest a large amount of energy while being comfortable to step on), received an impact force of 60 kg at different stepping frequencies (0.5, 1, 1.5, and 2 Hz). With a 0.1 mm thick PTFE layer, the peak voltage, current, and power were 79.28 V, 99.10 µA, and 7.86 mW (78.6 mW/mm), respectively, with an optimal load of 0.8 MΩ; while with a thickness of 0.2 mm, the same values were 120.78 V, 109.80 µA, and 13.26 mW (132.6 mW/mm), respectively, with an optimal load of 1.1 MΩ. The tile prototype equipped with a 0.2 mm thick PTFE layer illuminated a series of 100 to 150 LEDs. A list of small electronic devices that could be powered by the energy generated in both thickness cases is proposed (MCU + BLE and humidity, temperature, light, vibration sensors).

In [46], smart ceramic tiles (SCTs) based on TENG technology integrate electrodes with ceramic tiles using a layer-by-layer temperature gradient sintering method. Here, a contact force of 3 N was applied to the floor, a frequency of 2 Hz, and a travel distance of 4 cm was tested. Under these parameters, the measured open-circuit voltage stabilized at around 13 V, and the short-circuit current reached approximately 70 nA. Under a contact force of 3 N and a single-trip distance of 4 cm, as the frequency was increased from 1 Hz to 5 Hz, the open-circuit voltage remained relatively constant at 13 V, whereas the short-circuit current saw an increase from 40 nA (1 Hz) to 170 nA (5 Hz). This means a sensitivity of 40 nA/Hz and 34 nA/Hz.

In the triboelectric floor based on calcium silicate-carbon composite, different weight concentrations of SP up to 0.16 wt% were tested by applying a constant force of 10 N at a frequency of 5 Hz. The CS-SP TENG [47] doped with 0.04 wt% exhibits a maximum power density production of 2.13 W/m^2^ connected to a 1 MΩ resistance compared to 0.18 W/m^2^ of the CS-TENG without additives. The maximum values of V_PP_ = 110 V and I_PP_ = 9.8 µA of the CS-SP@ 0.04% are three times higher than those of the CS TENG (Figure 7e). Nonetheless, the electrical output (both the voltage and the dielectric constant) experienced a slight decline as the SP content exceeded 0.04%. The voltage produced increases with frequency (tested from 2 to 9 Hz), and power density with the applied load (tested from 2 to 10 N). The TENG can also charge commercial capacitors. Charging profiles for 10 µF and 47 µF capacitors by the CS-SP@ 0.04% TENG via a bridge rectifier, repeating the previous conditions, showed that they were successfully charged to 3.5 V and 2.2 V within 8 min, respectively. The electricity generated when the floor is repeatedly stepped on can charge a 100 µF capacitor to power a digital calculator while it discharges or to light up 100 green LEDs simultaneously.

### 4.2. Mechanical Disturbance Detection

Self-powered floors based on the triboelectric effect generate electrical signals from the mechanical energy they receive by TENGs, so many of them are referred to as motion sensors [47].

**Figure 8:**
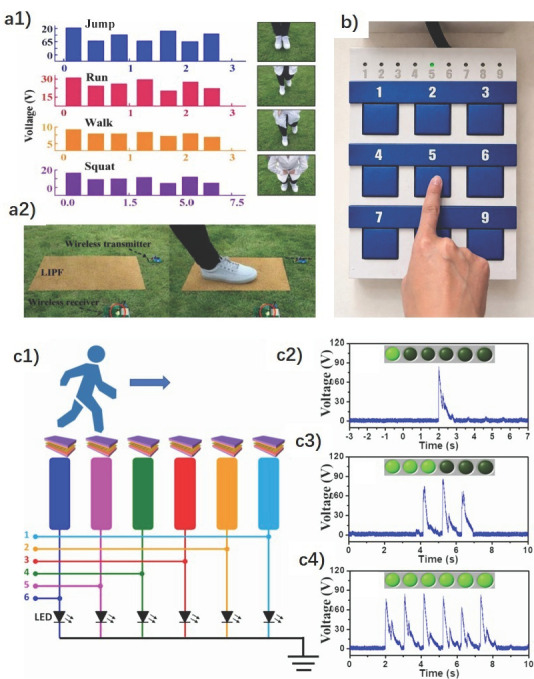
(**a**) Motion recognition and security alarm test on an LIPF. (**a1**) Output voltage of the LIPF resulted from human actions (squat, walk, run, jump) on the LIPF. (**a2**) The buzzer alarm system is triggered by stepping on the LIPF [42]. (**b**) Demonstration of the motion sensing application of the CSSP TENG [47]; (**c**) Self-powered u-TENG-based location-tracking system. (**c1**) Circuit diagram of the fabricated u-TENG-based tracking system. (**c2**–**c4**) Measured output voltage and real-time location mapping when the pedestrian arrives at the (**c2**) first, (**c3**) third, and (**c4**) sixth electrode [43]. AI was used to modify the original figures.

The corresponding shape of the electric signals, depending on different impact types, allows smart floors to perform activity recognition. For example, the FS-TENG [42] distinguishes between the waves produced by jumping, running, walking, and squatting (Figure 8a). The CS-SP TENG’s [47] ability to detect movement is demonstrated when a specific area of its surface is touched with a finger, causing an associated LED light to illuminate, indicating the finger’s position (with one light for each of the 9 possible regions), as shown in Figure 8b. Another self-powered location-tracking system was developed to count pedestrian volume and trace passenger movement in public areas, aiming to reduce energy consumption. Figure 8c represents the system that integrates u-TENGs [43] with six electrode channels, each assigned to a corresponding floorboard for real-time tracking. As pedestrians walked across the floorboards, voltage peaks were detected and triggered indicators on the monitor, revealing their location and making the μ-TENG-based floor useful as a self-powered tracking system for environmental monitoring and transportation control. LED bulbs connected to the floorboards emitted visible light signals, further indicating the pedestrian’s location. This design is suitable for low-traffic areas or public passages, particularly at night. The two aforementioned systems based on TENGs allowed for immediate display and observation of positional information without any power source device.

#### 4.2.1. Sports

Regarding the SF-TENG [37], its functionality as a motion sensor is also indicated. Its application is based on traditional wooden sports floors; the position of the ball can be identified, especially near the edges of the court. To confirm this, the voltage and current produced by the rolling of a roll of Scotch tape over the smart floor are analyzed. Depending on the duration of the signal, the motion and speed of the rolling tape can be noted.

**Figure 9:**
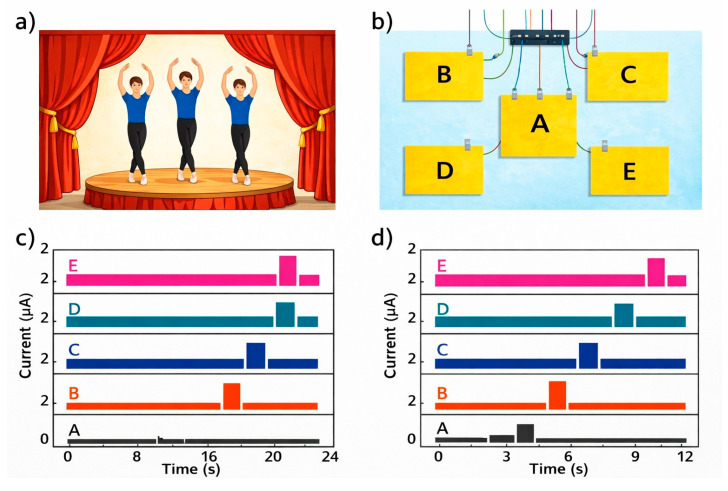
Application of the W-TENG in stage art. (**a**) Schematic diagram of dancers dancing in line on the stage. (**b**) Photo of W-TENG’s arrangement. (**c**) The real-time current signal output by the W-TENG when the dance shoes follow the path A-B-C-D-E. (**d**) The real-time current signal output by the W-TENG when the dance shoes follow the path AD-AB-AB-C-E [38]. AI was used to modify the original figures.

The smart floor, based on the W-TENG [38], can be installed on a stage to record and track the movement trajectory of the dancers (Figure 9). By following the dancers’ steps in real time, light effects can be created. For example, the lamps suspended overhead can illuminate the dancer’s trajectory in real time, with the dancers themselves being able to control the lights.

#### 4.2.2. Smart Home

The first TENG exposed mentioned its potential application for anti-theft devices [32]. In the home environment, the smart floor based on the SF-TENG [37] can detect falls in elderly individuals, monitor people’s movement to control the lighting, ventilation, and air conditioning, or detect unusual behaviors such as theft or break-ins.

**Figure 10:**
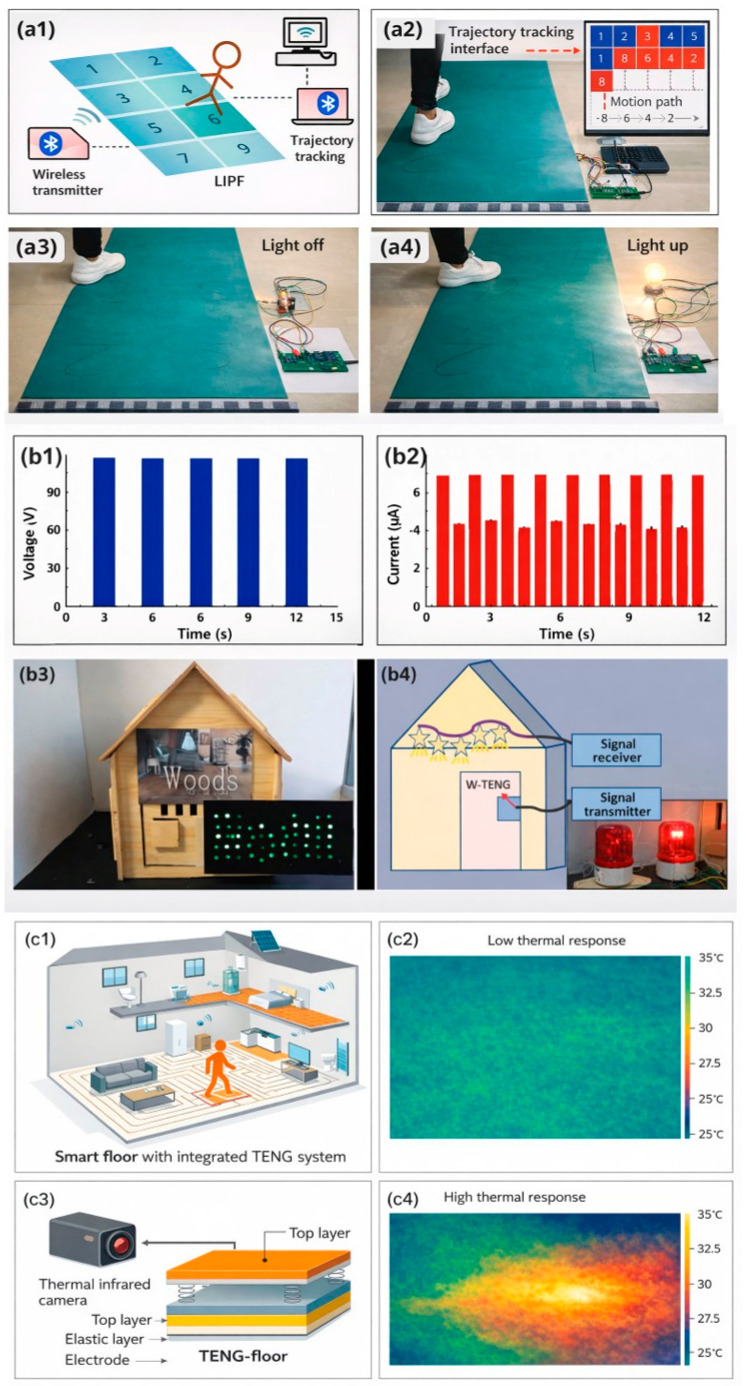
(**a**) LIPF trajectory tracking test and an intelligent switch demonstration. (**a1**) Trajectory tracking schematic diagram. (**a2**) Photograph of the trajectory tracking test as a person walks on the LIPF. (**a3**,**a4**) Photograph of the LIPF as an intelligent switch, triggering a lamp. (**b**) Applications of the W-TENG in the smart home. (**b1**) The open-circuit voltage and (**b2**) the short-circuit current of the W-TENG on the home door when pressed by hand. (**b3**) The W-TENG on the door driven by hand to light LEDs. (**b4**) Working principles of the W-TENG working as a sensor. (**b5**) Photo of the W-TENG applied for a light switch in smart homes. Inset: Photo of the house with the star lights on, which is controlled by the W-TENG. (**b6**) Photo of the W-TENG applied for a doorbell switch in smart homes. Inset: Photo of the bell that is on and is controlled by the W-TENG [38]. (**c1**) Schematic illustration of the self-heating TENG floor. (**c2**) Infrared image of the TENG floor at room temperature. (**c3**) Experimental set-up used to demonstrate the Joule heating generated by the TENG floor [44]. (**c4**) Infrared image of the TENG floor at higher temperature. AI was used to modify the original figures.

Reference is made to its use as a multifunctional self-powered motion sensor [42]. Three circuit diagrams were elaborated for security monitoring functions, trajectory tracking, and an intelligent switch. The first one aims to trigger a wireless buzzer system (applied, for example, to an alarm) by integrating the floor with a Bluetooth system and a voltage amplifier (Figure 10a). The second diagram is of a wireless transmitter, but in this case, the floor is divided into eight TENG modules. If one of the modules is stepped on, the corresponding one in the software interface changes color, mapping the route. The floor can be divided into other configurations regarding the desired trajectory coordinates of individuals to be tracked. The third diagram is of a wireless receiver functioning as an intelligent switch that turns a chip-type lamp on and off. Finally, satisfactory results are shown in air purification, demonstrating that the floor itself contributes to dust absorption and airborne bacteria sterilization. This was tested by comparing the aerial bacterial (*E. coli* density) levels of indoor air in the presence of PVC commercial flooring and a floor based on FS-TENG. After applying a periodic controlled 22 N force for 5 min by a human hand, 99.92% of the bacteria were inactivated across an area of 36 cm^2^. Electrostatic fields can attract and capture airborne particles. When these particles meet the electrostatic field generated by the FS-TENG, they become polarized and are drawn towards the surface, effectively removing them from the air. Micrographs showed that the FS-TENG floor exhibits much higher dust adsorption than the blank PVC floor.

The W-TENG [38] can be assembled as a smart light or a doorbell sensor (Figure 10b). The self-triggered system for both involves four elements: the TENG, the signal transmitter, the signal receiver, and lamps or a buzzer bell. The proposed light switch not only turns on when stepped on but also requires stepping specifically in the center. This fact can effectively save energy by avoiding the problem of longer lighting time than needed. Another potential use is mentioned as a hidden emergency sensor or alarm that requires no external power.

Various smart-home applications are proven with the FW-TENG [39], such as switching on a household lamp when someone walks on the floor or activating an electrochromic window by repeatedly pressing the TENG with the hand.

The wood-based TENG presented in [44] holds promise for application as self-heating sheets in intelligent building systems (Figure 10c). The viability of constructing a self-heating TENG floor by channeling the generated current through nickel-chromium alloy resistance wire to facilitate Joule heating was tested. Thermal imaging cameras were utilized to monitor the temperature distribution across the device surface during the heating process, and the results were positive. With a voltage of 240 V, a 100 × 100 mm^2^ sample could be heated from ambient temperature to 35.2 °C within 5 min. Other possible uses as energy suppliers for smart homes are mentioned, like self-triggered floor, switches or lighting systems, and energy storage.

The multi-material 3D-printed triboelectric floor presented in [41] shows TENG’s applicability as a miniature form of smart floor tile, which is responsive to foot pressure. An ATmega2560 microcontroller board captures a signal and makes a decision based on the threshold programmed in order to give alerts on the LCD display and buzzer. The MMP-TENG device (footprint area 2 × 4 cm^2^) generates a power of 0.89 µW by simple toe-tapping action. In addition, the C/PVDF|nylon 6-TENG generates a power density of 1.97 μW cm^−2^ and an energy density of 0.16 nJ cm^−2^ at 12 Hz, whereas the wood-based TENG floor provides a power density of 1.287 μW cm^−2^ and an energy density of 0.11 nJ cm^−2^ at 12 Hz. Additionally, a cyclic stability test was conducted for 10 h (over 360,000 cycles) to observe the long-term stability of the fabricated TENG device.

To facilitate a direct comparison among previously reported triboelectric floor systems, their material combinations, electrical output performance, operating conditions, and functional characteristics are summarized in Table 1. This comparative overview highlights the diversity of design strategies and performance metrics across different triboelectric floor configurations, providing a quantitative basis for the evaluation dimensions discussed in the following section.

#### 4.2.3. Healthcare

**Figure 11:**
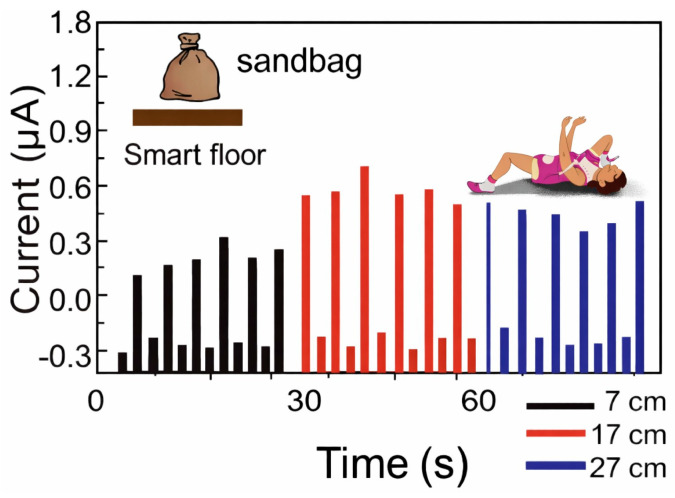
Demonstration of the SF-TENG as a fall detection sensor [37]. AI was used to modify the original figure.

Mode I of SF-TENG [37], with the aluminum balls enclosed inside shallow wood boxes, can harvest vibrational energy and, hence, detect sudden falls in elderly people who live alone. To test its capability as a nonintrusive fall-detection sensor, a 1 kg sandbag hits the floor from heights of 7, 17, and 27 cm (see Figure 11). The current pulse peak values generated ranged from 0.49 ± 0.05 to 0.8 ± 0.1 μA as the height increased, proving that the fall events would be indicated by current output variations.

With the FS-TENG [42], the possibility of using functions such as activity recognition for patients, children, or elderly monitoring is discussed. While the above studies demonstrate the broad applicability of triboelectric floors in healthcare monitoring, including fall detection and activity recognition, most reported systems rely on specific material combinations and structural designs tailored to sensing or harvesting functions. To further bridge the gap between literature-reported concepts and practical implementation, it is essential to experimentally validate alternative material systems and modular floor configurations under realistic excitation conditions [53].

In this context, an experimental triboelectric floor module based on fluorinated elastomer (FKM) and nitrile butadiene rubber (NBR) was designed and fabricated by the authors. The following section presents a systematic experimental validation of its electrical output characteristics, focusing on the influence of operating frequency and spatial electrode positioning within the floor module.

## 5. A High Electrical Efficiency Triboelectric Floor

**Figure 12:**
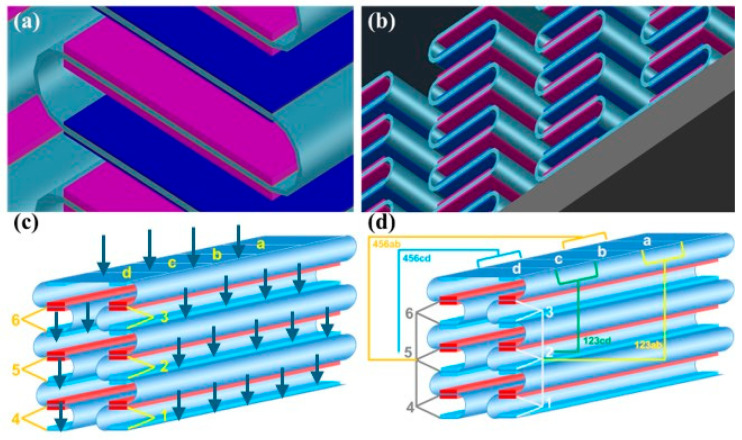
(**a**) TENG unit; (**b**) TENG strips embedded in the substrate; (**c**) TENG experimental schematic diagram; (**d**) Diagram of linkages at different points (123ab, 123cd, 456ab, 456cd). AI was used to modify the original figures.

A novel triboelectric floor composed of FKM as the electronegative layer and NBR as the electropositive layer is proposed in this work. Each tribolayer was laminated with a copper electrode on its backside and subsequently embedded into a flexible supporting substrate that holds multiple TENG strips. As shown in Figure 12, the prototype consists of four TENG units arranged in two spatial groups (positions 123 and 456), each characterized at two electrode points (ab and cd). And the experiment measured all four positions: 123ab, 123cd, 456ab, and 456cd (Figure 12d). This configuration was intentionally designed to evaluate the spatial non-uniformity of mechanical deformation and triboelectric charge generation within the module. The TENG floor strips (segments abcd) presented are 40 cm long and 4 cm wide with 4 × 4 cm^2^ TENGs placed one after the other along the strips.

All measurements were carried out using a PicoScope 7 system. A vertical cyclic load was applied manually to simulate repeated footstep-like contact, and two operating frequencies, 3 Hz and 6 Hz, were selected to represent slow and fast walking conditions. The applied force was kept as constant as possible during all tests. At each of the four electrode positions, both voltage and current-time waveforms were recorded, and the corresponding average (V_avg_, I_avg_) and peak (V_max_, I_max_) values were extracted for comparison.

Three TENG units with different tribolayer thicknesses were fabricated for preliminary comparison. During the experiments, the thicknesses of the triboelectric layers were kept constant in order to isolate the influence of excitation frequency and electrode position on the electrical output. Among the fabricated TENG units, the configuration with an NBR thickness of 1.5 mm and an FKM thickness of 1 mm exhibited the most stable and highest electrical output and was therefore selected as the representative configuration for detailed electrical characterization.

### 5.1. Voltage Output Characteristics

**Figure 13:**
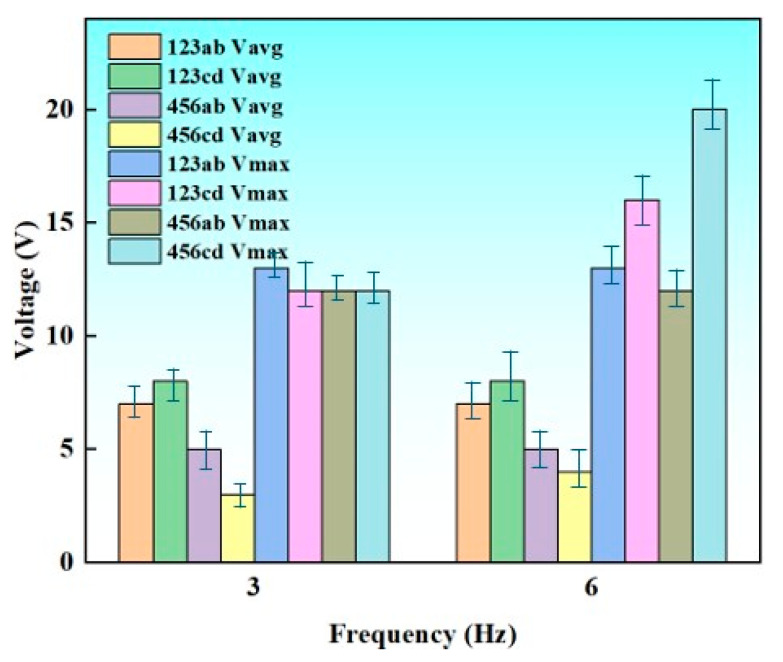
Voltage output characteristics of the FKM–NBR-based TENG floor module measured at different electrode positions under 3 Hz and 6 Hz excitation frequencies. AI was used to modify the original figure.

Figure 13 illustrates the output voltages measured at different electrode positions (123 and 456) and measurement points (ab and cd) under two operating frequencies (3 Hz and 6 Hz). Both the average output voltage (V_avg_) and the maximum instantaneous voltage (V_max_) were evaluated to analyze the operational performance of the fabricated TENGs.

At the overall level, increasing the operating frequency led to a noticeable improvement in the voltage output. V_max_ exhibited a strong frequency dependence. For example, the 456cd position showed the highest peak voltage, reaching approximately 20 V at 6 Hz, which is considerably higher than its value at 3 Hz (~12 V). This suggests that higher frequencies may induce stronger dynamic charge transfer, thereby contributing to elevated higher V_max_.

In contrast, the V_avg_ demonstrated relatively smaller variations across different frequencies and positions. For most locations in the 123 group, V_avg_ remained within the range of 5–8 V, while the 456 group showed slightly lower values (3–5 V). This indicates that the stable-state output of the system is less sensitive to the frequency change compared to the peak output, implying that the high-frequency enhancement primarily affects transient electrical behavior rather than steady-state performance.

Spatial variations were also evident. The 123 and 456 groups exhibited different voltage responses, with the 456 group generally producing higher V_max_ at 6 Hz. Moreover, within the same numerical group, distinct responses were observed between the ab and cd measurement points. For instance, the 456cd point demonstrated a higher V_max_ but a comparable or even lower V_avg_ relative to 456ab. This divergence suggests that local structural or contact differences at specific positions may influence instantaneous charge accumulation and release, contributing to position-dependent voltage behavior. The strong enhancement of V_max_ at specific positions under high-frequency excitation highlights potential resonance phenomenon, offering valuable insights for optimizing electrode configuration and system performance in future designs.

### 5.2. Current Output Characteristics

**Figure 14:**
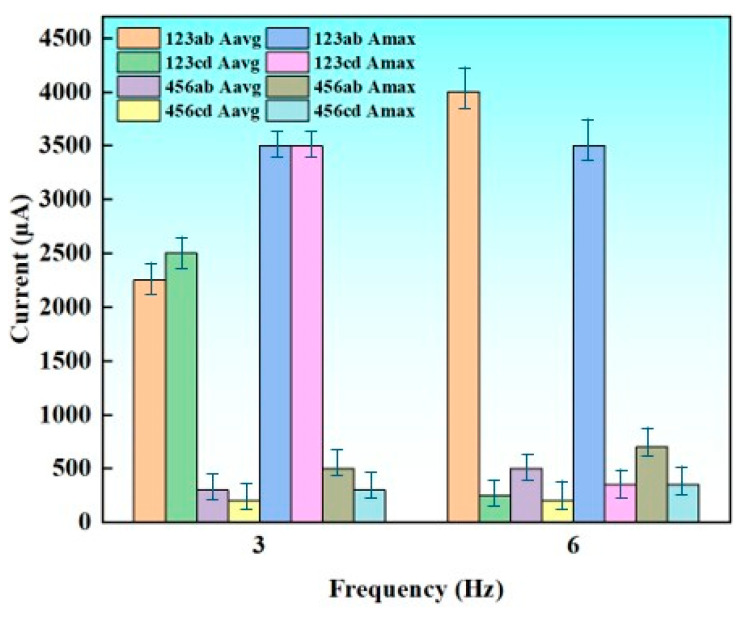
Current output characteristics of the FKM–NBR-based TENG floor module measured at different electrode positions under 3 Hz and 6 Hz excitation frequencies. AI was used to modify the original figures.

Figure 14 presents the average current (I_avg_) and maximum instantaneous current (I_max_) measured at different electrode positions (123 and 456) and measurement points (ab and cd) under excitation frequencies of 3 Hz and 6 Hz. Similar to the voltage outputs, the current responses exhibit a clear dependence on both frequency and spatial position.

At 3 Hz, the 123ab position produced the highest current output, with I_avg_ reaching approximately 2500 μA and I_max_ exceeding 4000 μA. This strong response indicates that this position provides more efficient charge transport during low-frequency operation. In contrast, the 456 group exhibited significantly lower current values at the same frequency range, with I_avg_ below 300 μA and I_max_ typically below 800 μA. These spatial differences suggest that electrode placement and local structural/environmental factors play a crucial role in governing current generation efficiency.

When the frequency increased to 6 Hz, the 123ab position continued to dominate with an even greater I_max_ value of nearly 4500 μA. Notably, other positions such as 123cd and 456ab also showed moderate increases in peak current at 6 Hz, indicating enhanced dynamic charge transfer under faster excitation. However, the enhancement was not uniform across all locations. For instance, 456cd displayed only a slight increase in both I_avg_ and I_max_, suggesting that this position may be less sensitive to frequency-induced mechanical stimulation or that local electrical pathways are less favorable for current amplification.

Comparing I_avg_ and I_max_, the peak current shows significantly stronger frequency dependence than the average current. This implies that the peak current exhibits high sensitivity to frequency, whereas average current remains relatively stable under various conditions.

Collectively, the data demonstrate that position 123ab exhibits the most efficient current-generation capability, while the 456 group shows comparatively weaker performance across both frequencies. For the three floor strips, each measuring 40 cm long x 4 cm wide and 1 mm thick, electrical current and voltage output were measured and found to be nearly 20 V and 4.5 mA respectively (0.1 W as maximum). The pronounced frequency-induced increase in I_max_ further suggests that higher operational frequencies can activate additional transient charge channels, offering a promising strategy for improving current output in future designs.

### 5.3. Discussion

The electrical measurements reveal two dominant tendencies: (i) the output increases with excitation frequency, and (ii) the performance varies significantly across spatial positions. The rise in Vmax and Imax at 6 Hz can be attributed to the shorter contact–separation cycle, which increases the number of charge–discharge events per unit time. This mechanism is commonly reported in contact-mode TENGs and is consistent with previous studies. This frequency-dependent behavior is consistent with previously reported contact–separation mode TENG floors based on polymeric and wood-based systems.

The strong response at position 123ab suggests better mechanical coupling or a larger effective contact area at this location. In contrast, the weaker output at 456ab and 456cd implies either reduced deformation, poorer material–surface contact, or higher local electrical impedance. These structural differences should be further investigated in future optimization.

Interestingly, the average voltage and current change much less than the peak values, indicating that steady-state charge transfer remains relatively stable, while the peak values are more sensitive to instantaneous mechanical input.

Overall, the results demonstrate that both frequency and spatial structure influence the device’s performance. Optimizing the floor’s internal mechanical alignment and ensuring uniform electrode contact could significantly improve the power-generation capability.

Beyond these factors, the configuration of each functional layer also plays an important role. In addition to material selection, the electrical performance of the proposed triboelectric floor is strongly influenced by the configuration of each functional layer, including the triboelectric layers, electrodes, and supporting substrate. Each layer contributes differently to charge generation, transport, and mechanical response under cyclic loading conditions.

In this work, fluorinated elastomer (FKM) and nitrile butadiene rubber (NBR) were used due to their significant differences in electronegativity and elastic behavior. These two materials have excellent electrical and mechanical properties that are needed to fabricate a floor on which people, when stepping, produce the necessary force to make both layers come into contact and generate energy. The main working energy nano-generation principle is the contact–separation mode [54] between triboelectric layers that is based on the electron transfer from the electronegative layer (FKM) to the electropositive one (NBR). This charge transport constitutes the electrical current conducted by the electrodes to the load.

In addition, the material with the highest Young’s modulus in relation to its fatigue strength and yield limit is liquid crystal polymer (LCP), which will be used as the substrate (see Figure S1). If a load of the same magnitude were applied to all twelve materials, LCP would experience less deformation and withstand that same load for a greater number of cycles than the others. With a yield limit value above 100 MPa, it is also the material that can return to its original state prior to loading with the heaviest applied load compared to the other materials: PVDF, ABS, PVC, PPS, PC, ABS + PC, PEI, PSU, PEI, PI and PCP (unfilled) (see Figures S2 and S3).

Using ANSYS [55], it was observed that the maximum working stress approaches the elastic limit of the LCP (120.83 MPa) as the load increases from 1500 N and exceeds it from approximately 2200 N onwards (see Figure S3).

In addition, ANSYS together with COMSOL allowed for the simulation of different electrical and mechanical properties. The energy generated was estimated at 50 W/m^2^, with energy efficiency presenting a high conversion rate of up to 1 W of power per step. In addition, the durability was 20 million steps, equivalent to an estimated lifespan of 5 years of use (or 10 million cycles at 50 MPa). The mechanical robustness of the triboelectric floor supports a maximum load of 1500–2220 N.

In particular, the thickness of the triboelectric layers plays a critical role in determining the output behavior. Thinner layers tend to enhance electrostatic induction and increase peak electrical output, while thicker layers improve mechanical stability and repeatability under footstep-like excitation. Based on the experimental observations reported in this work, an optimal thickness range can therefore be identified as a compromise between electrical performance and mechanical robustness, rather than a single extreme value. In our case, three floor strips measuring 40 cm long x 4 cm wide and 1 mm thick generated 0.1 W (20 V & 4.5 mA) of electrical power. This thickness-dependent trade-off provides a practical design guideline for future floor-integrated triboelectric nanogenerator systems [56]. An example of a prototype of the triboelectric floor described in this work can be seen in videos SV1 & SV2 and its design in photo S1. This prototype was presented at the EELISAxJOSEPHS Building Market-Ready Innovations (JOSEPHS Open Innovation Lab, 10–13 December 2024), Nürnberg, Germany). An electrical power of 4 W/m^2^ was calculated for this high-power efficiency floor.

## 6. Conclusions and Future Perspectives

This review of triboelectric nanogenerators focused on their use as floors not only refers to the materials used and the achieved electrical output values but also to the different functionalities and applications for which they have been designed. This analysis and synthesis of information from existing triboelectric floors aim to optimize them in various aspects, as exemplified below. The literature demonstrates progressive improvements occurring in TENG performance, such as the amount of electrical power produced, sustainability and environmental friendliness, weather resistance in non-favorable conditions, cost, and the exploration of new functions or domains to apply this innovative technology. In this case, it was observed that the implementation of TENGs in flooring constitutes an effective method for harnessing and recovering wasted energy from both animate and inanimate bodies moving daily over the ground. It is worth mentioning that the highest power density-producing floors (see Table 1) are the EMG-TENG, multifunctional PMNF-based TENG, and CS-TENG. This observation suggests that consolidating the use of TENGs in floors could involve combining triboelectricity with other types of energy harvesting (the highest electrical power density is achieved with an EMG-TENG hybrid device) or searching for new unconventional triboelectric materials (present in the multifunctional PMNF-based TENG and the CS-TENG). In these latter cases, the materials chosen for the electropositive layer were micro-nickel foam and ‘conductive cement’ (calcium silicate composites doped with Super P^®^ carbon black), both different from the non-biodegradable polymers more commonly used in triboelectric nanogenerators. This fact demonstrates that the pursuit of increased electrical performance is not incompatible with the use of more sustainable materials.

In addition to stepping, this floor could also be used for other activities such as vibration detection due to earthquakes or machinery operating in the street. Furthermore, frequency measurement may be used to determine the speed of cars between two points of detection or at only one point by detecting the passage of the two pair of wheels. This implies frequencies higher than 1 Hz (for 120 km/h, 33.3 Hz if the distance between wheels is of 1 m.

In addition, the experimental validation of an FKM–NBR-based triboelectric floor module confirms the feasibility of elastomer-based material systems for modular floor designs, offering insights into spatial optimization and frequency-dependent performance.

## Figures and Tables

**Table 1:** TENG-based floors described in previous literature.

TENG Name	Tribopositive Material	Tribonegative Material	Output Performance	Surface Contact Area	Power Density	Frequency, Force and Load	Other Parameters	Ref.
Multifunctional PMNF-based	PMNF	PDMS	V_OC_ = 187.8 V I_SC_ = 71.9 μA P = 9.3 mW S = 0.66 mW/Hz	50 × 50 mm = 25 cm^2^	3.7 W/m^2^	13.9 Hz, 3 MΩ	Conducting foam (Ni) + PDMS (colloidal + elastomer)	[33]
CNF-based TENG	CNFs	FEP	V_OC_ = 32.8 V I_SC_ = 35 μA P = 0.56 mW	40 cm^2^	−0.14 W/m^2^ *	-, -, 1 MΩ		[34]
SF-TENG	Mode I: Al Mode II: Rubber (Ex: shoe soles)	Mode I: PTFE Mode II: Wood	I: V_OC_ = 364 ± 43 V; I_SC_ = 9 ± 1 μA S = 109.2 μW/Hz II: V_OC_ = 238 ± 17 V; I_SC_ = 2.4 ± 0.3 μA	80 × 80 mm = 64 cm^2^ 195 × 167 mm ≈ 326 cm^2^	−18.2 mW/m^2^ 17.5 mW/m^2^	30 Hz	Mode I: C-S Mode II: Sliding	[35]
FS-TENG	Rubber (Ex: latex)	PVC	V_OC_ = 180 V I_SC_ = 7.5 μA	16 cm^2^	0.76 W/m^2^	5 Hz, 210 N, 30 MΩ		[36]
EMG-TENG	Al	Kapton + MoS_2_	TENG: V_OC_ = 350–500 V; I_SC_ = 20–38 μA EMG-TENG: V_OC_ = 1200 V; I_SC_ = 5 mA; P = 6 W.	TENG: 203.2 × 63.5 mm ≈ 129 cm^2^	E-T: 465 W/m^2^ *	TENG: 63.5 kg, 2 Hz E-T: Hands tapping	EMG-TENG: Nd_12_Fe_14_B magnets, Cu coils	[37]
W-TENG	Pine wood	PTFE	V_OC_ = 220 ± 20 V I_SC_ = 5.8 ± 0.5 μA	64 cm^2^	158.2 mW/m^2^	2 Hz, 50 MΩ	velocity of 0.352 m/s	[38]
u-TENG	Cu	PTFE	V_OC_ = 86 V I_SC_ = 10.8 μA P = 0.279 mW S = 0.056 mW/Hz	-	-	5 Hz, 500 N, 300 MΩ		[39]
FW-TENG	20 ZIF-8 @ spruce wood	PDMS @ spruce wood	V_OC_ = 24.3 V I_SC_ = 0.32 μA P = 7.3 mW	35 × 20 mm = 7 cm^2^	10.4 mW/m^2^	50 N, 300 MΩ		[40]
MMP-TENG	Nylon6	C/PVDF	V_OC_ = 0.7 V I_SC_ = 6 μA	60 × 30 × 0.3 mm^3^	197 μW/m^2^	2.8 N, 100 Ω, 7 Hz		[41]
W-TENG	O_2_ plasma-treated wood	C_4_F_8_ + O_2_ plasma-treated wood	V_OC_ = 227 V I_SC_ = 4.8 µA	100 × 80 mm = 8 cm^2^	18.86 mW/m^2^	180 N, 120 MΩ		[42]
Self-heating floor	Kapton	FEP	V_OC_ = 240 V I_SC_ = 550 nA	100 × 100 mm = 10 cm^2^	-			[43]
TEHFT	Al	PTFE	V_OC_ = 120.78 V I_SC_ = 109.80 μA P = 13.26 mW	300 × 300 mm = 900 cm^2^	0.15 W/m^2^ *	60 kg, 2 Hz, 1.1 MΩ		[44]
CS-SP TENG	CS composites + Super P^®^ carbon black	PTFE	V_pp_= 110 V I_pp_= 9.8 µA	16 cm^2^	2.13 W/m^2^	10 N, 5 Hz, 1 MΩ		[45]

-: not provided; * own calculation.

## Data Availability

Data will be made available on request.

## Supplementary Materials

The following supporting information can be downloaded at: https://www.mdpi.com/article/10.3390/s26072061/s1, Figure S1: (a) Vibrating table of CEDEZ and DAQ programs used for monitoring Lorca earthquake. (b) Different 3D components (planar NE and NS directions the same as Z one) of the seismic sensor; Figure S2: (a) Voltage generated by the 3D TENG seism sensor components. (b) Horizon-tal-to-Vertical Spectral Ratio (HVSR) and fundamental frequency of the site. (c) Smoothed HVSR curve and fundamental frequency of the site; Figure S3: Comparison of MAE with the (a) 1C, (b) 3C convolutional networks; Videos S1 and S2: Tribofloor operating in Nürnberg Josephs Open Innovation lab (2024).
