# Supplementary material for: A Comprehensive Review of Floor-Integrated Triboelectric Nanogenerators from Different Perspectives
Source: Sensors (Basel). 2026 Mar 25;26(7):2061. doi: 10.3390/s26072061 (PMC13075157; doi:10.3390/s26072061)
Supplement: Supplementary file 1 [file sensors-26-02061-s001.zip › sensors-4134280-supplementary.pdf]

SUPPORTING INFORMATION

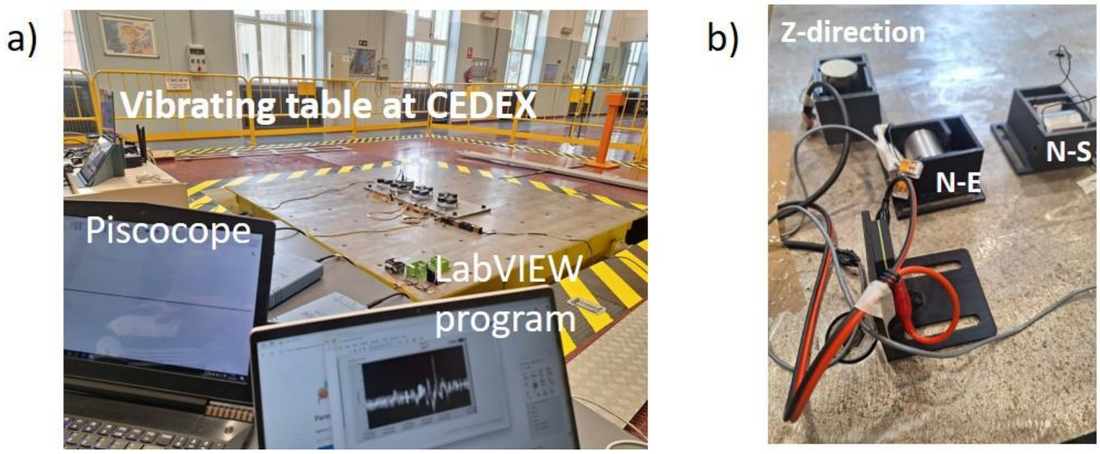

Figure S1. a) Vibrating table of CEDEZ and DAQ programs used for monitoring Lorca earthquake. b) Different 3D components (planar NE and NS directions the same as Z one) of the seismic sensor.

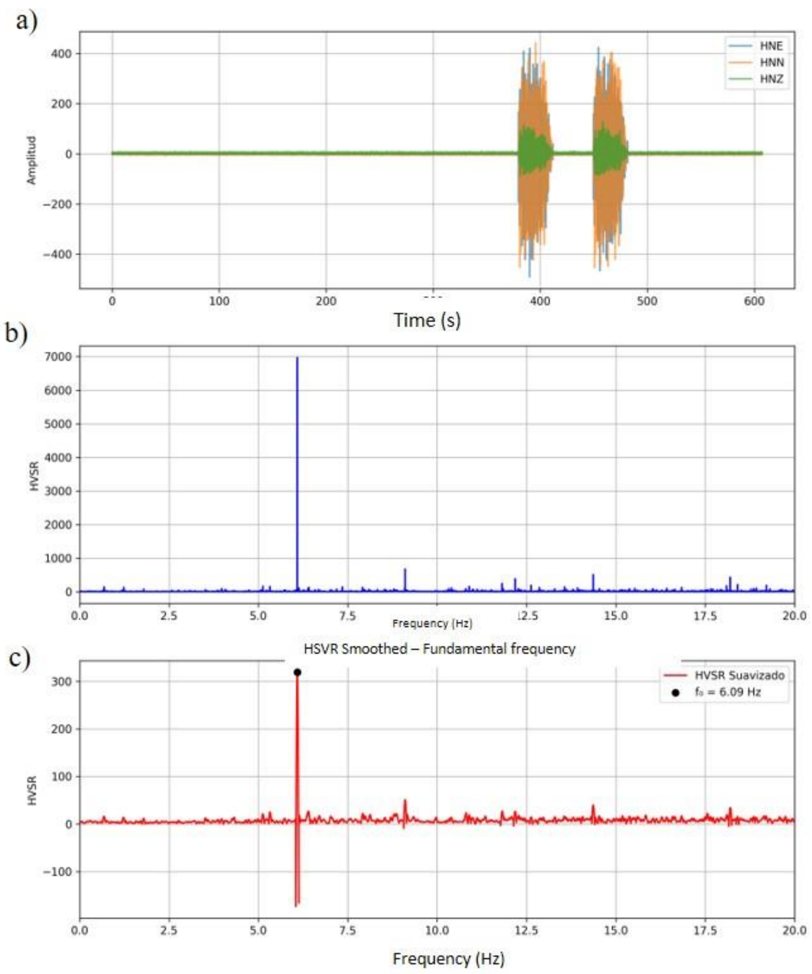

Figure S2. a) Voltage generated by the 3D TENG seism sensor components. b) Horizontal-to-Vertical Spectral Ratio (HVSR) and fundamental frequency of the site. c) Smoothed HVSR curve and fundamental frequency of the site.

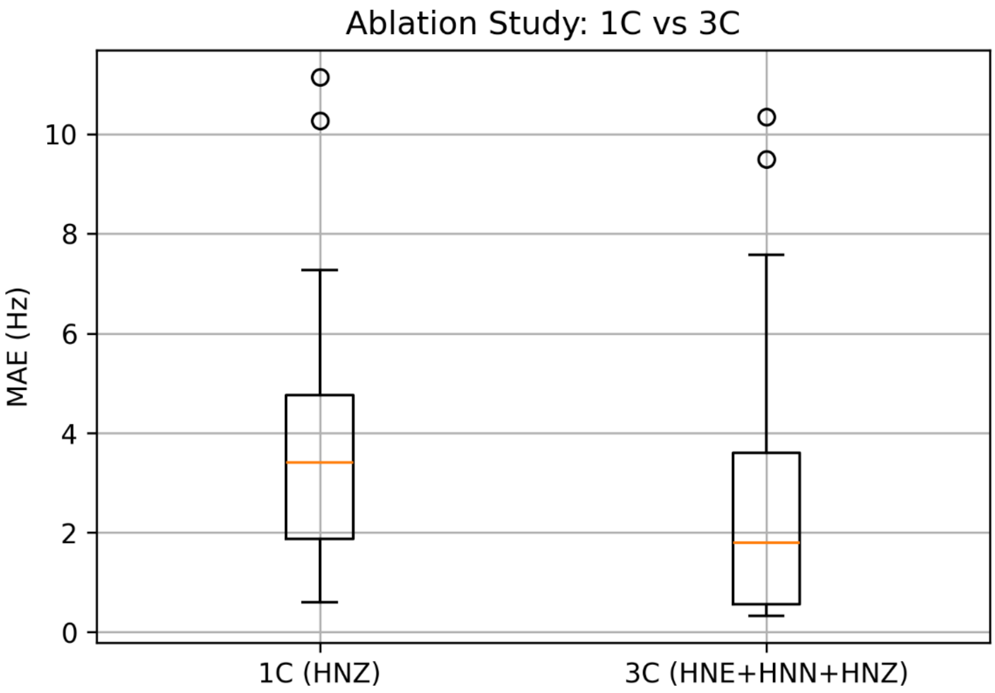

Figure S3. Comparison of MAE with the a) 1C, b) 3C convolutional networks.
